# Integrating multiple brain imaging modalities does not boost prediction of subclinical atherosclerosis in midlife adults

**DOI:** 10.1016/j.nicl.2022.103134

**Published:** 2022-07-29

**Authors:** Amy Isabella Sentis, Javier Rasero, Peter J. Gianaros, Timothy D. Verstynen

**Affiliations:** a Program in Neural Computation, University of Pittsburgh and Carnegie Mellon University, Pittsburgh, PA, USA; b Carnegie Mellon Neuroscience Institute, University of Pittsburgh and Carnegie Mellon University, Pittsburgh, PA, USA; c Department of Psychology, Carnegie Mellon University, Pittsburgh, PA, USA; d Department of Psychology, University of Pittsburgh, Pittsburgh, PA, USA; e Biomedical Engineering, Carnegie Mellon University, Pittsburgh, PA, USA

**Keywords:** Brain imaging, Prediction stacking, Intima-media thickness, Cardiovascular disease, CVD, cardiovascular disease, CA-IMT, carotid-artery intima-media thickness, FC, functional connectivity, CBF, cerebral blood flow, BOLD, blood oxygenation level dependent, FRS, Framingham Risk Score, SVR, support vector regression, RMSE, root mean squared error

## Abstract

**Background:**

Human neuroimaging evidence suggests that cardiovascular disease (CVD) risk may relate to functional and structural features of the brain. The present study tested whether combining functional and structural (multimodal) brain measures, derived from magnetic resonance imaging (MRI), would yield a multivariate brain biomarker that reliably predicts a subclinical marker of CVD risk, carotid-artery intima-media thickness (CA-IMT).

**Methods:**

Neuroimaging, cardiovascular, and demographic data were assessed in 324 midlife and otherwise healthy adults who were free of (a) clinical CVD and (b) use of medications for chronic illnesses (aged 30–51 years, 49% female). We implemented a prediction stacking algorithm that combined multimodal brain imaging measures and Framingham Risk Scores (FRS) to predict CA-IMT. We included imaging measures that could be easily obtained in clinical settings: resting state functional connectivity and structural morphology measures from T1-weighted images.

**Results:**

Our models reliably predicted CA-IMT using FRS, as well as for several individual MRI measures; however, none of the individual MRI measures outperformed FRS. Moreover, stacking functional and structural brain measures with FRS did not boost prediction accuracy above that of FRS alone.

**Conclusions:**

Combining multimodal functional and structural brain measures through a stacking algorithm does not appear to yield a reliable brain biomarker of subclinical CVD, as reflected by CA-IMT.

## Highlights

• Brain measures from MRI do not improve Framingham Risk Score prediction of CA-IMT.
• Prediction stacking is a flexible approach to determine added predictive utility.
• Multimodal stacking can be applied to individual difference factors.

## 1 Introduction:

Cardiovascular disease (CVD) encompasses many heart and vascular conditions that contribute to a primary cause of death for both men and women in the United States (Virani et al., 2021). Atherosclerotic coronary artery disease is the most common CVD, with 50% of Americans older than 45 (Bild et al., 2005, Virani et al., 2021) and 10% Americans ages 33–45 living with some form of subclinical disease that prestages later clinical conditions (Loria et al., 2007, Virani et al., 2021). In 2018, 13% of deaths in the United States were attributed to overt coronary artery disease (Virani et al., 2021). Numerous complications of atherosclerotic CVD, including ischemia and myocardial infarction, contribute to morbidity and mortality (Baber et al., 2015, Detrano et al., 2008).

Typically, CVD is not considered in relation to brain-based biomarkers. For example, most clinical diagnostic and assessment criteria, like the Framingham Risk Score (D’Agostino et al., 2008), focus on peripheral physiological factors, health behaviors (e.g., smoking), and demographics to predict someone’s risk of CVD (Elliott, 2007, King et al., 2012). Yet, there is cumulative evidence that structural and functional features of the brain associate with CVD risk factors and that CVD risk factors (e.g., blood pressure, lipid levels, etc.) may be precursors to neurocognitive decline, some dementias, and brain aging (Moroni et al., 2016, Srinivasa et al., 2016).

There are both efferent and afferent mechanisms by which brain structure and function can be linked to subclinical CVD. On the efferent or brain-to-body side, the brain systems for autonomic, neuroendocrine, and immune control shape peripheral physiology in ways that confer CVD risk (Gianaros and Wager, 2015, Tawakol et al., 2017). For instance, recent findings suggest the possibility that increased amygdala activity may increase hematopoietic tissue activity, which in turn leads to increased arterial inflammation and incident CVD events (Tawakol et al., 2017). There is also a large body of evidence supporting afferent or body-to-brain contributions as well, with longstanding evidence linking risk factors for CVD to premature brain aging, including cognitive decline (Marebwa et al., 2018, Srinivasa et al., 2016). Hence, it is well established that CVD is a risk factor for neurocognitive decline (O’Brien, 2006, Stampfer, 2006). Carotid-artery intima-media thickness (CA-IMT), a surrogate measure of preclinical atherosclerosis (Gianaros et al., 2020), is associated with risk factors for CVD (and cerebrovascular disease), including hypertension, diabetes and smoking (Crouse et al., 1996). Moreover, CA-IMT itself has been shown to associate with progressive cognitive decline (Wendell et al., 2009) and increased risk of dementia (Wang et al., 2021). In these regards, CA-IMT may plausibly reflect decreased perfusion of brain tissue as reflected by reduced CBF, which in turn can result in silent brain infarctions and microvascular damage as precursors to neurocognitive decline (Moroni et al., 2016).

In fact, many CVD outcomes, such as myocardial infarction and preclinical markers of CVD risk, have recently been associated with functional and structural features of macroscopic brain systems. Longitudinal studies, for example, suggest that baseline metabolic activity in the amygdala predicts future myocardial infarction and components of the metabolic syndrome (Tawakol et al., 2017), and that baseline levels of stress reactivity in the rostromedial prefrontal cortex are associated with future major adverse cardiovascular events (Moazzami et al., 2020). Moreover, structural MRI measures of brain aging (composite measures of ventricle size, sulcal size and white matter hyperintensities) and regional cerebral blood flow relate to individual differences in the magnitude of blood pressure lowering induced by antihypertensive medication (Jennings et al., 2008), as well as the longitudinal progression of blood pressure over multiple years (Jennings et al., 2017). Lastly, functional activation in insular, anterior cingulate, medial prefrontal, hypothalamus and brainstem regions, measured in response to mental stress and emotional stimuli, has been shown to predict clinical CVD events (Moazzami et al., 2020), mental stress-induced blood pressure reactivity (Gianaros et al., 2017), and CA-IMT (Gianaros et al., 2020).

It is also important to consider the influence of the cardiovascular system on the brain. For example, carotid artery stenosis, narrowing of the carotid artery usually due to atherosclerosis (plaque build-up), has been theorized to have a negative effect on cognitive function through reduced blood flow to the brain in asymptomatic cases and ischemic brain damage in symptomatic cases (Wang et al., 2016). Nickel and colleagues studied patients with high-grade carotid artery stenosis without ischemic brain lesions. Patients had lower cognitive function compared to controls, however there was no corresponding association with cortical thickness (Nickel et al., 2019). Cheng and colleagues also studied patients with asymptomatic carotid artery stenosis and found that patients displayed lower cognitive and memory performance than controls and this difference correlated with disruption in resting-state functional connectivity (FC) across multiple networks (Cheng et al., 2012). Thus, given the associations between cardiovascular system and neurocognitive systems, it should be possible to identify a reliable, predictive association between brain measures and preclinical markers of CVD.

At present, however, there is largely mixed evidence as to whether there are reliable functional and structural brain imaging correlates of subclinical markers of CVD, particularly indexed by CA-IMT. Functional evidence shows, for example, that CA-IMT is associated with higher regional cerebral blood flow in some areas (medial frontal gyrus, putamen, and hippocampal regions), but also lower regional cerebral blood flow in other areas (lingual, inferior occipital, and superior temporal regions) (Sojkova et al., 2010). Other findings indicate that CA-IMT associates with lower cerebral blood flow (CBF) in gray matter and across the entire brain (Cermakova et al., 2020). This association with CBF is particularly interesting given ongoing work showing that variability in CBF is detectable in the resting blood oxygenation level dependent (BOLD) signal measured with functional MRI (fMRI). For example, work by Fukunaga and colleagues (2008) utilized the ratio between BOLD signal activation and cerebral blood perfusion to demonstrate that resting-state activity incorporates both a neuronal or metabolic component as well as a vascular component (i.e., blood flow; (Fukunaga et al., 2008)). Furthermore, cerebral perfusion has been shown to correlate with resting-state BOLD signal and connectivity in terms of spatial distribution across the brain (Viviani et al., 2011). Since CA-IMT is associated with CBF, and CBF is associated with the resting state BOLD signal, this would appear to support the possibility of detecting associations across individuals in the variability of CA-IMT as related to the resting BOLD signal itself.

Separately from functional neuroimaging studies, there is structural brain imaging evidence indicating that CA-IMT is inversely associated with total brain tissue volume, as well as cortical tissue volume more specifically (Muller et al., 2011, Tuo et al., 2018). In parallel, however, other lines of evidence suggest no association between CA-IMT and total brain tissue volume or gray matter tissue volumes (Cermakova et al., 2020). Lastly, some structural neuroimaging findings suggest an inverse association of CA-IMT and cortical thickness (Cardenas et al., 2012), but again not all findings are consistent with the latter observations (Alhusaini et al., 2018). This heterogeneity in functional and structural brain imaging findings, as well as the isolated (unimodal) treatment of functional and structural brain imaging measures have created an open question as to whether the simultaneous (multimodal) modeling of functional and structural brain features would combine to predict a known marker of subclinical CVD and predictor of future clinical events; namely, CA-IMT. Moreover, whether such multimodal modeling would add to the prediction of subclinical CVD beyond established demographic, behavioral, and biological risk factors is unknown.

To elaborate, a majority of studies on the brain correlates of CVD risk, particularly CVD markers such as CA-IMT, use conventional analytical approaches that include univariate correlation and regression methods. A problematic feature of these methods is that they are not combined with out-of-sample validation testing, limiting inferences about model and sample generalizability. Moreover, these studies have historically relied on brain measures from a single neuroimaging modality, e.g., task-based or resting-state fMRI, structural connectivity, metabolic activity via PET. Such unimodal analyses do not exploit or account for the distinct neurobiological properties of different neuroimaging modalities, that when combined may improve predictive power. Lastly, a focus thus far on the brain correlates of CVD risk has been on particular neural systems or networks, rather than all systems and networks across the entire brain. Taken together, it appears that integrating and combining whole-brain modalities into a transmodal machine learning model (Rahim et al., 2016, Wolpert, 1992) has the potential to overcome methodological limitations to improve the predictive utility and robustness of putative brain biomarkers of CVD risk to facilitate replication and generalization.

In the above regards, an effective biomarker or multimodal brain correlate of CVD risk would have the following characteristics. First, it would take into account the unique variability inherent to the different measures derived from imaging modalities (e.g., cortical thickness, cortical surface area, and tissue volumes derived by structural MRI, as well as dynamic activity measures reflecting neural networks derived by fMRI). Second, it would rely on either standard clinical brain imaging sequences (e.g., T1 weighted anatomicals) or MRI data acquisition sequences that are amenable to clinical contexts and testing in diverse populations of people (e.g., resting-state fMRI). Third, it would reliably predict CVD risk, not just associate with it (e.g., out of sample validation testing). Finally, a reliable brain correlate of CVD risk would account for additional variability above-and-beyond that already accounted for by other established risk factors for CVD. To these ends, the present study examined whether morphological and basic functional measures derived from T1-weighted and resting-state fMRI data could be combined in a multimodal machine learning analysis framework (with predictor variables comprised of cortical surface area, cortical thickness, subcortical volumes and whole-brain resting-state FC) to reliably predict inter-individual variability in CA-IMT in a sample of neurologically healthy adults. For this we modified an identical multimodal machine learning approach used previously to predict “brain age” (Liem et al., 2017) – a measure of brain aging when compared to chronological age that has been shown to correlate with numerous risk factors of CVD, including smoking and diabetes (Cole, 2020). We then evaluated performance against the prediction of CA-IMT by Framingham Risk Score (D’Agostino et al., 2008).

## 2 Materials and methods

### 2.1 Participants

Neuroimaging, cardiovascular, and demographic data were collected from N = 324 healthy participants (ages 30–51, 49% female) from the Pittsburgh Imaging Project (see Table 1). All participants provided informed consent. The University of Pittsburgh Human Research Protection Office granted study approval. Detailed information about the study population has been published in Gianaros et al., 2020. This is the first report bearing on the multimodal prediction of CA-IMT from this sample and these results have not been published previously. Data and code are available at https://github.com/CoAxLab/multimodal-imt.

**Table 1:** Sample characteristics (N = 324; 164 Men, 160 Women).

Characteristic	Mean or (%)	SD
Age (years)	40.30	6.28
Race (%) Caucasian African-American Multiracial/ethnic	66.00 28.40 5.60	
BMI (kg/m^2^)	26.93	5.07
Smoking status (%) Never Former Current	62.65 20.06 17.28	
Seated resting systolic BP (mm Hg)	120.80	10.01
Seated resting diastolic BP (mm Hg)	72.63	8.75
Seated resting HR (bpm)	74.10	9.63
Glucose (mg/dL)	88.34	9.75
HDL (mg/dL)	50.73	16.06
Triglycerides (mg/dL)	94.43	56.94
CA-IMT (mm)	0.61	0.08
FRS	5.35	5.99

Note: SD = standard deviation, BMI = body mass index, BP = blood pressure, HDL = high-density lipoproteins, CA-IMT = carotid artery intima-media thickness, FRS = Framingham Risk Score.

### 2.2 Preclinical atherosclerosis

**Fig. 1:**
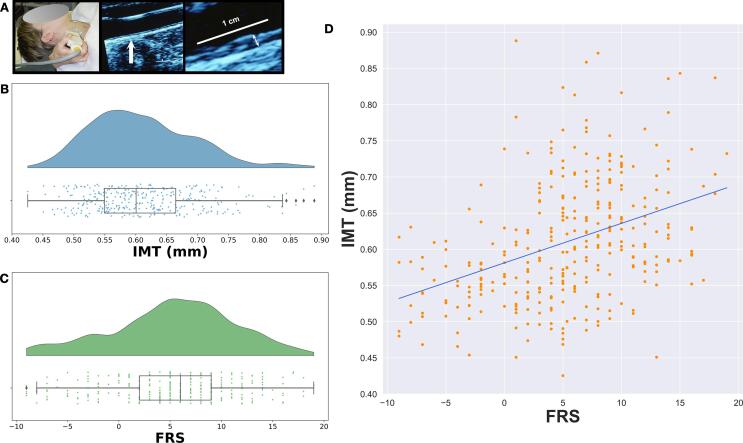
A) Left panel shows CA-IMT acquisition using ultrasound. Middle and right panels show example ultrasound images with the CA-IMT indicated. B) Raincloud plot showing distribution of CA-IMT (mm) in our sample. C) Raincloud plot showing distribution of FRS in our sample. D) Scatterplot showing the linear regression of FRS on CA-IMT. Line of best fit shown in blue. CA-IMT = carotid artery intima-media thickness, FRS = Framingham Risk Score.

Carotid-artery IMT was measured at three locations (distal common carotid artery, carotid artery bulb, and internal carotid artery) by trained ultrasound sonographers using an Acuson Antares ultrasound device (Acuson-Siemens, Malvern, PA). Measurements were obtained on both the left and right carotid artery in three specific locations: 1) both the near and far walls of the distal common carotid artery, located 1 cm proximal to the carotid bulb (the location at which the near and far walls of the common carotid are no longer parallel and extending to the flow divider), 2) far wall of the carotid bulb, and 3) the first centimeter of the internal carotid measuring from the distal edge of the flow divider. These three measurements were then averaged bilaterally and across locations to calculate the mean CA-IMT, which was used as the outcome variable. Further information about measurement methods and test-retest reliability of CA-IMT measurements can be found in Gianaros et al., 2020. Fig. 1A shows example images of IMT acquisition. Fig. 1B shows the CA-IMT values in our sample, which are approximately normally distributed (Liu et al., 2019, Polak et al., 2011).

### 2.3 Framingham risk

Framingham Risk Score (FRS) was calculated for each participant according to D’Agostino et al. (2008). This metric incorporates age, sex, smoking, hypertension and cholesterol data from each participant. Five participants had missing FRS data. For analysis purposes, these missing values were imputed using the mean FRS. Fig. 1C shows the distribution of FRS.

### 2.4 MRI data acquisition and processing

Functional blood oxygenation level-dependent images were collected on a 3 Tesla Trio TIM whole-body scanner (Siemens), equipped with a 12-channel phased-array head coil. Resting-state functional images were acquired over a 5-minute period with eyes open and the following acquisition parameters: FOV = 205×205 mm, matrix size = 64×64, TR = 2000 ms, TE = 28 ms, and FA = 90°. Thirty-nine slices (interleaved inferior-to-superior, 3 mm thickness, no gap) were obtained for each of 150 volumes (three initial volumes were discarded to allow for magnetic equilibration). T1-weighted neuroanatomical magnetization prepared rapid gradient echo (MPRAGE) images were acquired over 7 min 17 sec with the following parameters: FOV = 256×208 mm, matrix size = 256×208, TR = 2100 ms, inversion time = 1100 ms, TE = 3.31 ms, and FA = 8° (192 slices, 1 mm thickness, no gap).

Resting-state fMRI data were preprocessed using SPM12 and included slice-timing correction, realignment to the first image using a six-parameter rigid-body transformation, co-registration to skull-stripped and biased-corrected MPRAGE images, normalization to standard Montreal Neurological Institute (MNI) space and smoothing using a 6 mm full-width-at-half-maximum (FWHM) Gaussian kernel. Head motion at the individual participant image level was estimated via framewise displacement (FD) according to Power et al., 2015 for use during FC processing (described further below).

Resting-state data were denoised, including six motion parameters, white matter (WM), cerebrospinal fluid (CSF), and global signal (GS). The first principal component for each of WM, CSF and GS was used. Data were also bandpass filtered with a range of 0.009–0.08 Hz. A functional correlation matrix was calculated using the Craddock 200 parcellation (Craddock et al., 2012) by first computing the average time series from the voxels within each of the 200 parcels, and then calculating the z-transformed Pearson correlation coefficient between pairs of parcel time series. The upper triangular elements were extracted from the functional correlation matrix to form a vector of 19,900 FC features for each participant. FD was regressed out and the final FC vector for each participant is comprised of the resultant residuals. By this approach, correlations that are partial for FD between all possible ROIs in the Craddock atlas.

MPRAGE images were analyzed using FreeSurfer (v6), with 148 cortical thickness and cortical surface area measures from the *thickness* and *area* freesurfer files respectively, using the Destrieux Atlas (Destrieux et al., 2010), as well as 67 subcortical volume measures directly extracted from the *aseg.stats* freesurfer file of each participant.

### 2.5 Multimodal prediction of IMT

We adopted a transmodal approach to stacking learning for prediction of CA-IMT (Rahim et al., 2016, Wolpert, 1992). In machine learning, stacking is classified as an ensemble learning method and involves combining predictions from a set of models into a new meta feature matrix for subsequent input into a new model for final prediction (Liem et al., 2017, Rasero et al., 2021).

**Fig. 2:**
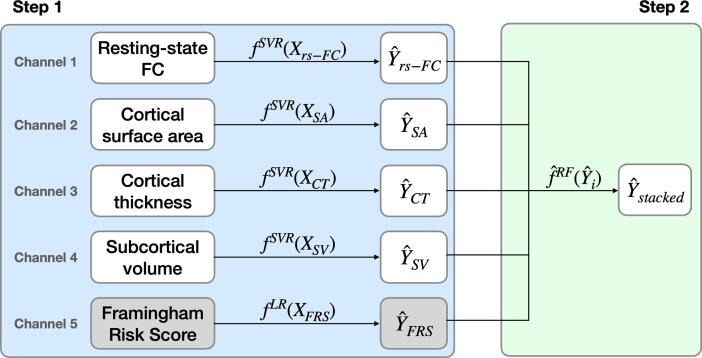
Prediction stacking model schematic, with linear SVR and linear regression used in the unimodal predictions and random forest used in the multimodal prediction. FC = functional connectivity, SVR = support vector regression, LR = linear regression, RF = random forest.

As detailed in Fig. 2, our model comprised a two-step process that used multiple output predictions for each participant from a first level support vector regression (SVR) model as the inputs into the second level random forest model. The set of first level SVR models used different groups of features, or channels, corresponding to 1) resting-state FC, 2) cortical surface area, 3) cortical thickness and 4) subcortical volume measures. Performance of the predictive models at the first and second levels of analyses was determined using cross-validation. This model was predicated on the work of Liem et al., 2017, who used this transmodal approach to predict brain age. In order to validate our model implementation, we predicted brain age in our sample and compared the results to those presented in Liem et al., 2017.

To do so, we first split the data such that 80% were used for training and the remaining 20% for testing. Next, we used five-fold cross-validation during the training stage to generate out-of-sample SVR predictions for each channel on the training set data. We used a previously tuned parameter, C, for this type of data from Liem et al., 2017. As input into the second level, the out-of-sample predictions from the training set as well as the test set predictions were stacked across channels, forming new matrices of 80% observations × 4 channels and 20% observations × 4 channels, respectively. The second level random forest model was then tuned for the tree depth hyperparameter and trained using five-fold cross-validation to generate out-of-sample predictions on the new training matrix and tested on the new test matrix to generate the final predictions for brain age. Performance of the single-channel and stacked models was then evaluated by comparing participant’s chronological age with the participant’s predicted brain age in the out-of-sample test data. Prediction error was measured using the coefficient of determination, R-squared, and the root mean squared error (RMSE). All predictive analyses were performed using scikit-learn (Pedregosa et al., 2011).

Once validated using age, this analysis pipeline was used to predict CA-IMT as the target outcome variable. An additional fifth channel consisting of a participant’s FRS was included in this pipeline. Since over parameterization is not a concern with a single feature model, simple linear regression (LR) was used for the single channel prediction of CA-IMT from FRS. Thus, five single channels (four brain measures plus FRS) were stacked as input into the second level random forest model. Performance was similarly evaluated through comparison of observed CA-IMT values with the predicted CA-IMT values in the out-of-sample test data.

We subsequently evaluated and compared model performance on CA-IMT prediction for every possible combination of single data channels, again using the coefficient of determination, R-squared, representing model goodness-of-fit as the measure of model performance.

Finally, in order to test robustness of our analysis and confirm that results were not dependent on a particular training/testing data split, we generated 100 random training/testing splits, using different random seeds, for analysis through our cross-validated, channel combination implementation. Final model performance was evaluated using the median of the Pearson correlation coefficient, coefficient of determination, RMSE and Bayesian information criterion (BIC) values of each partition.

## 3 Results

We first tested whether we could confirm previously reported patterns in our data set. Our primary outcome measure, mean CA-IMT, was measured using ultrasound (Fig. 1A; see Materials and Methods 2.2). Consistent with the assumptions of our statistical models, these CA-IMT values across our sample were approximately normally distributed (Fig. 1B), with a slight skew, in ranges consistent with an unbiased sample across the population (Stein et al., 2008). We next wanted to replicate the well established relationship between FRS and CA-IMT (Polak et al., 2010, Riccio et al., 2006). FRS values were approximately normally distributed (Fig. 1C). As expected the linear regression of the association between FRS and CA-IMT confirms a positive association, with a Pearson correlation coefficient of r = 0.3857, p < 0.001 (Fig. 1D). Taken together, these results are in line with the literature and constitute a replication of effects shown previously (DuPont et al., 2021, Polak et al., 2010, Riccio et al., 2006).

**Fig. 3:**
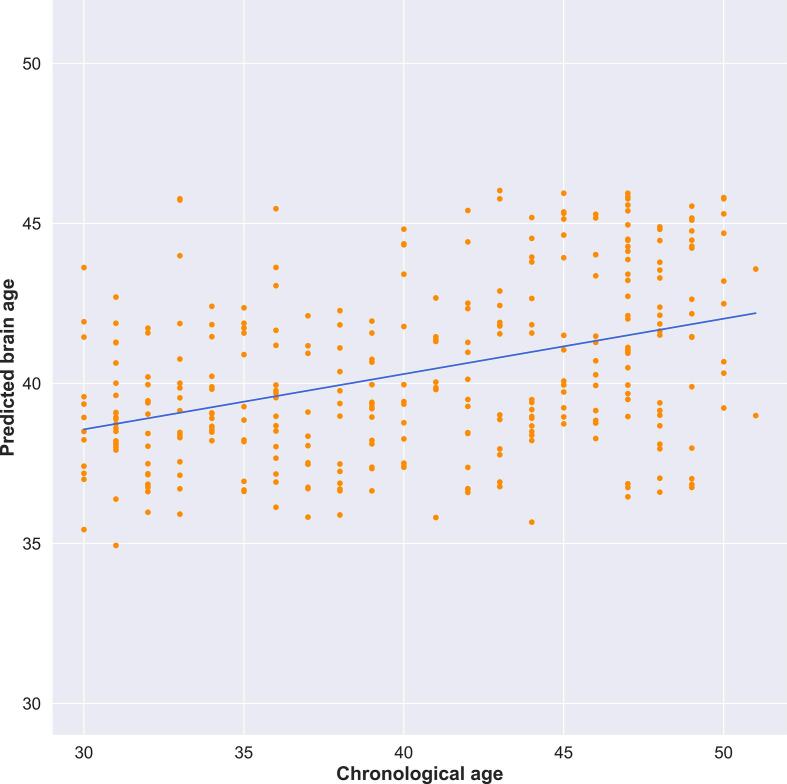
Scatterplot showing correlation between participants’ chronological age and predicted brain age according to multimodal model. Blue line represents the line of best fit.

In order to validate the feasibility of our transmodal stacking approach, we first attempted to replicate the findings of Liem et al., 2017 and predict chronological age using morphological brain measures as well as resting-state FC. This prior study was able to predict chronological age from the same imaging measures used here, with an accuracy of +/- 4 years. Implementing our own version of the pipeline, applied it to our sample, revealed an association between chronological age and predicted brain age that was positive and equivalent in magnitude to the original report, with a Pearson’s correlation coefficient of r = 0.5246, p < 0.001 and coefficient of determination, R-squared = 0.2732 for the hold-out test set. Fig. 3 shows the observed versus predicted scatter plot for chronological age and brain age. Our age prediction error (∼4 years) approximated that of the results presented in Liem et al., 2017, confirming the validity of our stacking approach.

**Fig. 4:**
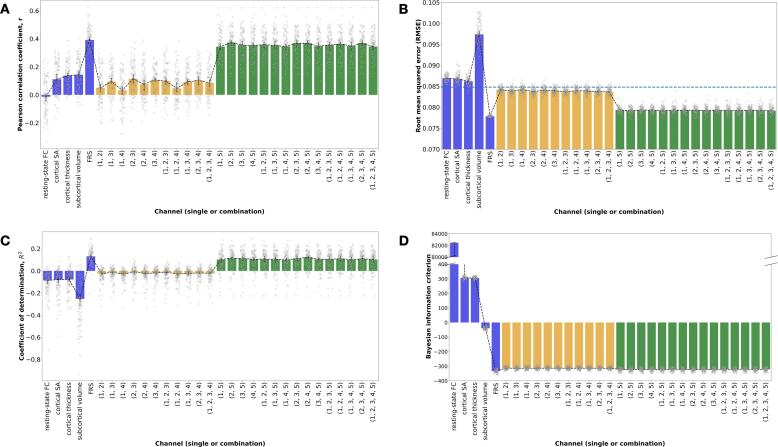
For all panels, blue bars show single channel predictions of CA-IMT. Yellow bars show channel combination predictions that include only brain measures. Green bars show channel combinations predictions that include FRS. Error bars indicated 95% confidence intervals (calculated using 1000 bootstrap iterations). Channel combinations are indicated numerically with 1 = resting-state FC, 2 = cortical SA, 3 = cortical thickness, 4 = subcortical volume, 5 = FRS. Median values for the Monte Carlo simulation for single channel and every possible channel combination prediction of mean CA-IMT: A) Pearson correlation coefficient, r, B) RMSE (horizontal dotted line represents the standard deviation of CA-IMT in our sample, 0.084 mm), C) coefficient of determination, and D) Bayesian information criterion. CA-IMT = carotid-artery intima-media thickness, FRS = Framingham Risk Score, FC = functional connectivity, SA = surface area.

In order to evaluate our primary aim of determining whether clinically obtainable brain imaging measures boost the prediction accuracy of individual differences in markers of CVD risk, we applied our stacked learning approach to predicting CA-IMT. Fig. 4 shows the distribution of four different metrics for each random Monte Carlo data partition for both the single channel predictions of CA-IMT, as well as every possible channel combination for the second level random forest prediction of CA-IMT. Panel A shows Pearson correlation coefficients, r values, panel B shows RMSE values (with the horizontal dotted line representing the standard deviation of CA-IMT in our sample, 0.084 mm), panel C shows coefficient of determination, R-squared values, and panel D shows BIC values.

Across all panels, the blue bars show the first level SVR and linear regression CA-IMT predictions using the single channel brain measures and FRS. Fig. 4A shows that FRS has the largest median Pearson correlation coefficient, r = 0.3916 (95% CI [0.3739, 0.4058]), with the brain measures showing much smaller associations (resting-state FC median r = -0.0132 (95% CI [-0.0375, 0.0096]), cortical SA median r = 0.1111 (95% CI [0.0959, 0.1402]), cortical thickness median r = 0.1373 (95% CI [0.1237, 0.1560]), subcortical volume median r = 0.1435 (95% CI [0.1245, 0.1646])). Fig. 4B demonstrates that the single channel predictions of CA-IMT from the brain measures had the largest median RMSE values (resting-state FC median RMSE = 0.0869 mm, cortical SA median RMSE = 0.0868 mm, cortical thickness median RMSE = 0.0862 mm, subcortical volume median RMSE = 0.0.0974 mm), and were higher than the standard deviation of CA-IMT (Fig. 4B). The single channel FRS prediction of CA-IMT had the lowest RMSE out of all models, with a median RMSE = 0.0778 mm, and was the only single channel model with an RMSE value beneath the standard deviation of CA-IMT in our sample. Fig. 4C shows that median R-squared values for the single channel brain measure predictions of CA-IMT are all negative, indicating that our model does not appropriately predict CA-IMT using brain measures. However, the median R-squared value for single channel prediction of CA-IMT using FRS is positive, R-squared = 0.1314 (95% CI [0.1139, 0.1421]), indicating that FRS accounts for over 13% of the variance in CA-IMT. Fig. 4D shows a large range of BIC values for the single channel predictions of CA-IMT, with FRS being the most negative (resting-state FC median BIC = 82,454, cortical SA median BIC = 306.18, cortical thickness median BIC = 305.06, subcortical volume median BIC = -38.99, FRS median BIC = –332.89). Note that BIC reflects the amount of information lost by a model, so lower values are better. This confirms the results from panels A-C, demonstrating that the FRS single channel model is preferred over the single channel brain measure models.

In all panels, the yellow bars show the second level random forest CA-IMT predictions from the channel combinations comprised of brain measures only. Fig. 4A shows the median Pearson correlation coefficients, which ranged between r = 0.0328 (95% CI [0.0025, 0.0467]) and r = 0.1143 (95% CI [0.0963, 0.1383]). Stacking only the brain measures did not improve performance accuracy over the best single channel brain measure. Fig. 4B shows the RMSE values, which hovered around the standard deviation of CA-IMT, and slightly improved upon the RMSE values of the single channel brain measures. Fig. 4C shows that median R-squared values for the channel combination predictions of CA-IMT using only brain measures are all negative, albeit less negative than the R-squared values from the single channel brain models. This indicates that our model does not appropriately predict CA-IMT using brain measures. Fig. 4D shows improved median BIC values for the channel combination predictions of CA-IMT using only brain measures compared to that of the single channel brain measure models, ranging from BIC = -312.89 to BIC = -314.22. However, these BIC values do not improve upon the median BIC value from the single channel FRS model, indicating that a combination of brain measures will not be a better feature selection choice than FRS.

In all panels of Fig. 4, the green bars show the second level random forest CA-IMT predictions from the channel combinations that include FRS. Individually, some brain measures perform above chance in predicting CA-IMT, specifically the morphometry measures from T1, when looking at the correlation between observed and predicted values. However, the effect size is smaller compared to that of the single channel FRS model. In Fig. 4A, the median predicted vs. observed correlation values for the channel combinations that include FRS were more than three times that of the maximum value of the channel combinations that only include brain measures, ranging between r = 0.3436 (95% CI [0.3261, 0.3653]) and r = 0.3727 (95% CI [0.3412, 0.3772]). Fig. 4B shows that the inclusion of FRS resulted in a reduction in median RMSE values, hovering around 0.079 mm. Fig. 4C demonstrates positive median R-squared values for the channel combination predictions of CA-IMT that include FRS, ranging between R-squared = 0.09 (95% CI [0.0731, 0.1051]) and R-squared = 0.11 (95% CI [0.0810, 0.1131]), though all are lower than that of the single channel FRS model. Similarly, Fig. 4D shows median BIC values that are smaller than that of the channel combinations that only include brain measures, but larger than that of the single channel FRS model, ranging between BIC = -320.90 and BIC = –322.23. Adding in FRS resulted in an overall increase in performance across all metrics shown in Fig. 4. However, this is solely driven by FRS, as none of the channel combinations that include FRS perform better than FRS alone. These results indicate that brain measures do not assist in the prediction of CA-IMT beyond FRS.

## 4 Discussion

Our goal for this study was to evaluate whether structural and functional brain measures from standard, clinically accessible MRI scans (T1 and resting-state fMRI) could be used to boost prediction of a marker of preclinical CVD above what is achievable from more standard clinical metrics, namely the FRS. Results show that our stacking algorithm is a sound methodology. We also see a strong association between FRS and CA-IMT, as expected. By comparison, we fail to find an improvement in our model predictions when using these brain measures individually, or in combination.

Our findings emphasize the complex nature of the role of the brain in CVD risk. Emerging mechanistic insights that link markers of CVD risk with structural and functional brain measures provide support for the need to further understand the role of the brain in CVD risk. Subclinical markers of CVD risk have been shown to associate with cerebral hypoxia and silent brain infarctions (Qiu and Fratiglioni, 2015). CVD risk factors (such as smoking, diabetes, obesity, hypertension) also demonstrate associations with inflammation, oxidative stress, brain atrophy, ischemic changes and reduced CBF, which can also contribute to neurocognitive decline (de Bruijn and Ikram, 2014, Knopman et al., 2011, Pase et al., 2012, Song et al., 2020, Williamson et al., 2018). In addition, there is emerging evidence that functional and structural alterations within the brain, particularly in brain systems for peripheral physiological control, may confer CVD risk via efferent or brain-to-body pathways (Gianaros and Wager, 2015). Thus, based on this evidence in the literature, we predicted CA-IMT could connect with resting-state fMRI in two ways (body-to-brain and brain-to-body pathways), yet failed to detect an association.

Alignment of our work with prior literature is seen in a few different ways. Firstly, we replicate existing findings that demonstrate a substantial relationship between FRS and CA-IMT (DuPont et al., 2021, Polak et al., 2010, Riccio et al., 2006). Secondly, our methodological approach replicates that of Liem et al., 2017 in a new sample, validating stacked learning as a useful tool for predicting individual differences from MRI-based measures. Finally, our results confirm some of the findings in the neuroimaging literature, namely that individually, cortical thickness and brain volumes are associated with CA-IMT (Cardenas et al., 2012, Muller et al., 2011, Tuo et al., 2018). However, these associations are weak in comparison to that of FRS and do not add to that model’s predictive power. Our findings also contrast with prior literature showing no association between CA-IMT and structural brain measures, including cortical thickness and brain volumes (Alhusaini et al., 2018, Cermakova et al., 2020), though it is possible that differences in the demographic makeup of the sample populations preclude direct comparisons.

Our failure to detect a reliable prediction of CA-IMT from the sole functional measure, resting-state FC, contrasts with recent work from our group showing reliable prediction of CA-IMT using task-based fMRI measures (Gianaros et al., 2020). This contrast is particularly revealing. Resting-state FC is a passive measure reflecting global intrinsic brain networks (Friston, 1994, Salvador et al., 2005). Thus, targeted recruitment of specific brain networks during stressful or engaging tasks is likely necessary in order to use such functional brain signals as a predictor of individual differences in CA-IMT (Finn, 2021). Indeed, this type of task-based functional brain measure could boost the predictive power of FRS. However, there is a vast body of tasks that needs to be explored before this type of functional data can be incorporated into our stacking model. Furthermore not all task-based fMRI work has observed an association between CVD risk factors and neural activity patterns (e.g., there is no association between the cortico-limbic network activation during a social threat fMRI task and an individual’s cardiometabolic risk (Lederbogen et al., 2018)). Finally, it is possible that alternative resting-state FC analysis methods, including dynamic resting-state FC, graph analysis metrics (global efficiency, degree centrality), or wavelet methods for determining connectivity, may provide different measures of the underlying resting hemodynamic response which may include signal for detecting individual differences in CA-IMT. However, a full survey of these different methods and their relationship to CA-IMT is itself an entire study in its own right, constituting a promising next step in investigation.

One interpretive consideration regarding the findings of this study centers on the particular model used and whether it is truly effective for using multimodal brain measures to predict CA-IMT. Notably, we first replicated the method exactly by successfully predicting brain age in our sample, demonstrating that the method works as expected. We also see above chance prediction performance from individual brain measures as well as FRS. Finally, we showed successful stacking with FRS, despite no performance improvements when we include brain measures.

Another important consideration when interpreting our findings relates to our sample population. It is possible that the study selection criteria may have restricted the range of subclinical CVD present in the sample, which could partly explain the failure of multimodal brain measures to predict CA-IMT. We note, however, that FRS explained a moderate amount of the variance in CA-IMT across individuals (see Fig. 4). Notwithstanding, a useful future direction would be to replicate and extend our approach in a more diverse sample, spanning a range of preclinical and clinical phenotypes of CVD.

It is also possible that predictive performance in the present study was limited by the use of CA-IMT, which has been suggested to have limited performance in the prediction of clinical CVD outcomes (Goldberger et al., 2010, Lorenz et al., 2012). Nevertheless, evidence from intervention trials indicates that CA-IMT progression is an important outcome measure, especially for the detection of early pathophysiological vascular changes (Hodis et al., 2016). Moreover, it has been noted that carotid ultrasound is feasible in nearly all persons, relatively inexpensive, and associated with the incident (future) development of atherosclerotic plaques (Peters et al., 2012, Tschiderer et al., 2020). In these regards, CA-IMT is regarded as a surrogate measure of the atherosclerotic disease process that predicts later CVD events (Baber et al., 2015, Øygarden, 2017, Peters et al., 2012, Wendell et al., 2017). Taken together, while CA-IMT has advantages as a subclinical CVD marker, it is possible that predictive performance from MRI measures could be improved by using other subclinical disease markers, such as coronary calcium scores or omnibus metrics based upon CA-IMT, such as arterial stiffness and endothelial function, which reflect vascular morphology and function (Kobayashi et al., 2004).

The brain imaging modalities we used may have further constrained predictive performance, creating the possibility that other imaging modalities may capture brain features that are more reliably associated with subclinical CVD (e.g., arterial spin labeling for the assessment of cerebral blood flow and diffusion imaging for the assessment of white matter morphology) (Jennings et al., 2013).

In addition, our cross-sectional findings do not rule out the possibility that baseline brain measures could forecast future (prospective) changes in disease endpoints, as has been found previously. Baseline amygdalar activity has been shown to predict future occurrence of CVD events (Tawakol et al., 2017), changes in visceral adipose tissue (Ishai et al., 2019) as well as risk of Takotsubo syndrome (Radfar et al., 2021). Levels of stress reactivity within the rostromedial prefrontal cortex are also associated with future adverse CVD events (Moazzami et al., 2020).

## 5 Conclusions

In summary, the present cross-sectional human neuroimaging findings suggest that subclinical CVD reflected by CA-IMT does not reliably relate to a combined brain biomarker generated by stacking functional and structural features of the brain. Rather, CA-IMT predicted by FRS alone outperformed aggregate and individual MRI measures. In these regards, combining multimodal functional and structural brain measures by prediction stacking may not have utility in otherwise healthy midlife adults to characterize the neural correlates of subclinical CVD indexed by CA-IMT.

## Declaration of Competing Interest

The authors declare that they have no known competing financial interests or personal relationships that could have appeared to influence the work reported in this paper.
