# Supplementary material for: Integrating multiple brain imaging modalities does not boost prediction of subclinical atherosclerosis in midlife adults
Source: Neuroimage Clin. 2022 Jul 29;35:103134. doi: 10.1016/j.nicl.2022.103134 (PMC9421527; doi:10.1016/j.nicl.2022.103134)
Supplement: Supplementary data 1 [file mmc1.docx]

**Supplementary table 1:** *Distribution of features for each imaging modality.*

Mean and standard deviation across the sample for each feature for every imaging modality. Modalities are separated into different excel sheets. rs_FC = resting-state functional connectivity; anat_SA = cortical surface area; anat_thickness = cortical thickness; anat_vol = subcortical volume.
