# Supplementary material for: Integrating multiple brain imaging modalities does not boost prediction of subclinical atherosclerosis in midlife adults
Source: Neuroimage Clin. 2022 Jul 29;35:103134. doi: 10.1016/j.nicl.2022.103134 (PMC9421527; doi:10.1016/j.nicl.2022.103134)
Supplement: Supplementary data 2 [file mmc2.docx]

**Supplementary Table 2a:** *Pearson correlation coefficient values and one-sided p-values for single channel Monte Carlo data partitions.*

For each single channel, the Pearson correlation coefficient values and one-sided p-values were calculated. MC = Monte Carlo, FC = functional connectivity, SA = surface area, FRS = Framingham Risk Score.

| **MC data partition** | **resting-state FC** | **cortical SA** | **cortical thickness** | **subcortical volume** | **FRS** |
| --- | --- | --- | --- | --- | --- |
| 1 | 0.0848 (p=0.2528) | 0.2329 (p=0.0320) | 0.1306 (p=0.1519) | 0.0251 (p=0.4220) | 0.4087 (p=0.0004) |
| 2 | 0.0107 (p=0.4666) | 0.2600 (p=0.0190) | 0.2011 (p=0.0556) | 0.2552 (p=0.0209) | 0.4516 (p=0.0001) |
| 3 | 0.1016 (p=0.2121) | 0.0019 (p=0.4940) | 0.0732 (p=0.2828) | 0.1177 (p=0.1772) | 0.5623 (p<0.0001) |
| 4 | -0.1173 (p=0.1779) | -0.0961 (p=0.2250) | 0.0273 (p=0.4151) | 0.1513 (p=0.1163) | 0.2869 (p=0.0108) |
| 5 | 0.1317 (p=0.1497) | 0.2264 (p=0.0360) | 0.2611 (p=0.0186) | 0.0284 (p=0.4117) | 0.4068 (p=0.0004) |
| 6 | 0.2547 (p=0.0211) | 0.0609 (p=0.3164) | 0.0404 (p=0.3755) | 0.1267 (p=0.1591) | 0.3187 (p=0.0051) |
| 7 | -0.0103 (p=0.4679) | 0.0890 (p=0.2420) | 0.0228 (p=0.4291) | 0.1543 (p=0.1118) | 0.3016 (p=0.0077) |
| 8 | -0.0158 (p=0.4508) | -0.0714 (p=0.2875) | 0.1262 (p=0.1602) | 0.0190 (p=0.4408) | 0.4076 (p=0.0004) |
| 9 | -0.0117 (p=0.4634) | 0.0055 (p=0.4827) | 0.1176 (p=0.1774) | 0.0053 (p=0.4834) | 0.3550 (p=0.0020) |
| 10 | -0.0211 (p=0.4342) | 0.2362 (p=0.0301) | 0.0522 (p=0.3411) | 0.2561 (p=0.0205) | 0.4496 (p=0.0001) |
| 11 | -0.0659 (p=0.3025) | 0.2178 (p=0.0419) | 0.2345 (p=0.0311) | -0.0284 (p=0.4120) | 0.3019 (p=0.0077) |
| 12 | -0.1050 (p=0.2046) | 0.2582 (p=0.0197) | 0.1489 (p=0.1202) | 0.0679 (p=0.2971) | 0.4307 (p=0.0002) |
| 13 | 0.2229 (p=0.0383) | 0.0243 (p=0.4246) | 0.0174 (p=0.4458) | 0.1634 (p=0.0984) | 0.3343 (p=0.0035) |
| 14 | -0.0054 (p=0.4830) | 0.0223 (p=0.4305) | 0.2120 (p=0.0463) | 0.1100 (p=0.1935) | 0.5037 (p<0.0001) |
| 15 | 0.0928 (p=0.2329) | 0.1131 (p=0.1868) | 0.1155 (p=0.1817) | 0.3043 (p=0.0072) | 0.4586 (p=0.0001) |
| 16 | -0.0580 (p=0.3245) | 0.2053 (p=0.0518) | 0.3137 (p=0.0058) | 0.1900 (p=0.0663) | 0.2069 (p=0.0505) |
| 17 | 0.0143 (p=0.4555) | 0.1201 (p=0.1722) | 0.2269 (p=0.0357) | 0.2902 (p=0.0100) | 0.4777 (p<0.0001) |
| 18 | -0.0088 (p=0.4725) | 0.2554 (p=0.0208) | 0.1204 (p=0.1717) | 0.2254 (p=0.0367) | 0.3065 (p=0.0069) |
| 19 | -0.1390 (p=0.1366) | -0.0253 (p=0.4212) | 0.1240 (p=0.1644) | 0.0046 (p=0.4855) | 0.2755 (p=0.0138) |
| 20 | -0.0416 (p=0.3721) | 0.0096 (p=0.4701) | 0.1472 (p=0.1228) | 0.1173 (p=0.1781) | 0.4906 (p<0.0001) |
| 21 | 0.1184 (p=0.1758) | 0.2112 (p=0.0469) | 0.2187 (p=0.0413) | 0.1413 (p=0.1327) | 0.3492 (p=0.0023) |
| 22 | -0.2008 (p=0.0559) | 0.0934 (p=0.2314) | 0.1552 (p=0.1104) | 0.2158 (p=0.0434) | 0.3807 (p=0.0010) |
| 23 | 0.0787 (p=0.2682) | 0.0363 (p=0.3879) | 0.1372 (p=0.1399) | 0.1410 (p=0.1333) | 0.3916 (p=0.0007) |
| 24 | -0.1491 (p=0.1198) | 0.0998 (p=0.2163) | -0.0094 (p=0.4705) | -0.0754 (p=0.2768) | 0.3293 (p=0.0039) |
| 25 | 0.1547 (p=0.1111) | 0.0037 (p=0.4884) | 0.2038 (p=0.0532) | 0.1457 (p=0.1254) | 0.4428 (p=0.0001) |
| 26 | 0.0722 (p=0.2855) | 0.3047 (p=0.0072) | 0.1231 (p=0.1662) | 0.1177 (p=0.1772) | 0.3106 (p=0.0062) |
| 27 | -0.1303 (p=0.1525) | 0.0419 (p=0.3711) | 0.2965 (p=0.0087) | 0.1687 (p=0.0914) | 0.2935 (p=0.0093) |
| 28 | -0.0617 (p=0.3140) | 0.1068 (p=0.2004) | 0.0272 (p=0.4154) | 0.2586 (p=0.0195) | 0.4234 (p=0.0002) |
| 29 | 0.1364 (p=0.1412) | 0.0402 (p=0.3762) | 0.2327 (p=0.0321) | 0.1486 (p=0.1206) | 0.4788 (p<0.0001) |
| 30 | 0.1235 (p=0.1654) | 0.0322 (p=0.4002) | 0.0048 (p=0.4851) | -0.0530 (p=0.3388) | 0.3424 (p=0.0028) |
| 31 | -0.1358 (p=0.1424) | 0.0776 (p=0.2712) | 0.0582 (p=0.3240) | 0.0717 (p=0.2868) | 0.3810 (p=0.0009) |
| 32 | 0.1517 (p=0.1158) | 0.1273 (p=0.1581) | 0.1649 (p=0.0964) | 0.2121 (p=0.0462) | 0.4093 (p=0.0004) |
| 33 | 0.2337 (p=0.0316) | -0.1750 (p=0.0834) | 0.1964 (p=0.0599) | 0.0520 (p=0.3416) | 0.4242 (p=0.0002) |
| 34 | 0.1327 (p=0.1479) | 0.0718 (p=0.2864) | 0.2684 (p=0.0160) | 0.2526 (p=0.0220) | 0.3069 (p=0.0068) |
| 35 | 0.1376 (p=0.1391) | 0.0978 (p=0.2210) | 0.1436 (p=0.1288) | 0.1027 (p=0.2097) | 0.4660 (p=0.0001) |
| 36 | -0.0364 (p=0.3876) | -0.0315 (p=0.4025) | 0.1090 (p=0.1957) | 0.3137 (p=0.0058) | 0.5166 (p<0.0001) |
| 37 | -0.0428 (p=0.3685) | 0.1347 (p=0.1444) | 0.1549 (p=0.1108) | 0.2246 (p=0.0372) | 0.3164 (p=0.0054) |
| 38 | 0.0131 (p=0.4591) | 0.1091 (p=0.1954) | 0.2400 (p=0.0280) | 0.0060 (p=0.4813) | 0.4319 (p=0.0002) |
| 39 | -0.1476 (p=0.1222) | 0.2280 (p=0.0350) | 0.2544 (p=0.0213) | 0.3112 (p=0.0062) | 0.4782 (p<0.0001) |
| 40 | -0.0060 (p=0.4811) | -0.0973 (p=0.2221) | 0.1289 (p=0.1549) | 0.1733 (p=0.0855) | 0.3470 (p=0.0025) |
| 41 | -0.1553 (p=0.1103) | 0.1941 (p=0.0621) | 0.2447 (p=0.0257) | 0.3280 (p=0.0041) | 0.4904 (p<0.0001) |
| 42 | -0.0150 (p=0.4530) | 0.0126 (p=0.4608) | 0.1703 (p=0.0893) | 0.2897 (p=0.0101) | 0.4125 (p=0.0004) |
| 43 | -0.1061 (p=0.2021) | 0.3205 (p=0.0049) | 0.0802 (p=0.2642) | 0.2017 (p=0.0550) | 0.4753 (p<0.0001) |
| 44 | -0.0442 (p=0.3643) | 0.1372 (p=0.1398) | 0.0673 (p=0.2985) | 0.0810 (p=0.2623) | 0.3135 (p=0.0058) |
| 45 | 0.0967 (p=0.2237) | 0.1709 (p=0.0885) | -0.0033 (p=0.4896) | -0.0507 (p=0.3452) | 0.3860 (p=0.0008) |
| 46 | -0.0506 (p=0.3457) | 0.0910 (p=0.2372) | 0.1581 (p=0.1060) | 0.1081 (p=0.1975) | 0.3738 (p=0.0012) |
| 47 | 0.0862 (p=0.2491) | 0.2311 (p=0.0331) | 0.0763 (p=0.2746) | 0.1337 (p=0.1461) | 0.4906 (p<0.0001) |
| 48 | 0.0900 (p=0.2397) | 0.0761 (p=0.2750) | 0.0125 (p=0.4610) | 0.1630 (p=0.0991) | 0.4050 (p=0.0005) |
| 49 | -0.0124 (p=0.4614) | 0.2661 (p=0.0168) | 0.1384 (p=0.1377) | 0.2693 (p=0.0157) | 0.3545 (p=0.0020) |
| 50 | 0.0228 (p=0.4291) | 0.1425 (p=0.1307) | 0.1374 (p=0.1394) | 0.2915 (p=0.0097) | 0.3587 (p=0.0018) |
| 51 | 0.0313 (p=0.4029) | -0.1180 (p=0.1765) | 0.2490 (p=0.0236) | 0.1206 (p=0.1712) | 0.4323 (p=0.0002) |
| 52 | -0.0537 (p=0.3367) | 0.2314 (p=0.0329) | 0.0498 (p=0.3480) | 0.1737 (p=0.0849) | 0.2858 (p=0.0110) |
| 53 | -0.0246 (p=0.4234) | 0.2020 (p=0.0547) | 0.1608 (p=0.1022) | 0.1759 (p=0.0822) | 0.2952 (p=0.0089) |
| 54 | -0.1245 (p=0.1635) | 0.1890 (p=0.0674) | 0.1912 (p=0.0650) | 0.1406 (p=0.1339) | 0.3872 (p=0.0008) |
| 55 | -0.1357 (p=0.1425) | 0.1798 (p=0.0776) | 0.1717 (p=0.0875) | 0.2242 (p=0.0375) | 0.4271 (p=0.0002) |
| 56 | -0.0194 (p=0.4395) | 0.2832 (p=0.0117) | 0.0952 (p=0.2271) | 0.0292 (p=0.4093) | 0.4293 (p=0.0002) |
| 57 | 0.0365 (p=0.3874) | 0.0592 (p=0.3211) | -0.0017 (p=0.4946) | 0.2570 (p=0.0202) | 0.2564 (p=0.0204) |
| 58 | -0.2077 (p=0.0498) | 0.2451 (p=0.0255) | 0.2602 (p=0.0189) | -0.0144 (p=0.4550) | 0.4934 (p<0.0001) |
| 59 | -0.2348 (p=0.0309) | 0.0711 (p=0.2882) | 0.1320 (p=0.1492) | 0.1538 (p=0.1125) | 0.3456 (p=0.0026) |
| 60 | -0.2185 (p=0.0414) | 0.2958 (p=0.0088) | 0.1478 (p=0.1219) | 0.2090 (p=0.0487) | 0.4690 (p<0.0001) |
| 61 | -0.3372 (p=0.0032) | 0.0865 (p=0.2485) | 0.1756 (p=0.0826) | 0.0735 (p=0.2818) | 0.4545 (p=0.0001) |
| 62 | -0.0027 (p=0.4916) | 0.0800 (p=0.2648) | 0.0936 (p=0.2310) | 0.1303 (p=0.1524) | 0.2001 (p=0.0564) |
| 63 | -0.1180 (p=0.1766) | 0.2394 (p=0.0284) | 0.1531 (p=0.1136) | 0.2316 (p=0.0328) | 0.4060 (p=0.0004) |
| 64 | -0.0125 (p=0.4610) | -0.0181 (p=0.4436) | 0.0793 (p=0.2666) | 0.1019 (p=0.2114) | 0.3306 (p=0.0038) |
| 65 | 0.1382 (p=0.1380) | 0.1460 (p=0.1248) | 0.1047 (p=0.2051) | 0.1736 (p=0.0850) | 0.5750 (p<0.0001) |
| 66 | 0.0042 (p=0.4869) | 0.1728 (p=0.0861) | 0.3516 (p=0.0022) | 0.2334 (p=0.0317) | 0.4341 (p=0.0002) |
| 67 | -0.0138 (p=0.4569) | -0.1542 (p=0.1119) | 0.0413 (p=0.3729) | 0.2690 (p=0.0158) | 0.4552 (p=0.0001) |
| 68 | -0.0736 (p=0.2817) | 0.2692 (p=0.0157) | 0.2311 (p=0.0331) | 0.2733 (p=0.0145) | 0.3592 (p=0.0018) |
| 69 | 0.0463 (p=0.3583) | 0.2254 (p=0.0366) | 0.1512 (p=0.1165) | -0.0685 (p=0.2953) | 0.3020 (p=0.0077) |
| 70 | -0.2455 (p=0.0253) | 0.2582 (p=0.0197) | 0.0422 (p=0.3703) | 0.4068 (p=0.0004) | 0.6272 (p<0.0001) |
| 71 | -0.0833 (p=0.2564) | 0.0269 (p=0.4166) | 0.2323 (p=0.0323) | 0.1528 (p=0.1141) | 0.2878 (p=0.0105) |
| 72 | 0.0956 (p=0.2263) | 0.1662 (p=0.0946) | 0.2620 (p=0.0182) | 0.2482 (p=0.0240) | 0.4977 (p<0.0001) |
| 73 | -0.1050 (p=0.2044) | 0.2339 (p=0.0314) | 0.0961 (p=0.2250) | 0.2308 (p=0.0332) | 0.3772 (p=0.0011) |
| 74 | 0.1116 (p=0.1900) | 0.0684 (p=0.2956) | 0.1664 (p=0.0944) | 0.1545 (p=0.1115) | 0.4225 (p=0.0003) |
| 75 | 0.1469 (p=0.1233) | 0.1790 (p=0.0786) | 0.1688 (p=0.0912) | 0.0576 (p=0.3257) | 0.1906 (p=0.0657) |
| 76 | 0.1934 (p=0.0629) | 0.0865 (p=0.2483) | 0.0946 (p=0.2285) | -0.0518 (p=0.3421) | 0.4169 (p=0.0003) |
| 77 | 0.1686 (p=0.0915) | 0.0449 (p=0.3623) | 0.1288 (p=0.1553) | 0.0919 (p=0.2350) | 0.4457 (p=0.0001) |
| 78 | 0.1059 (p=0.2025) | 0.0676 (p=0.2977) | 0.0562 (p=0.3296) | 0.0549 (p=0.3333) | 0.2178 (p=0.0419) |
| 79 | 0.1310 (p=0.1510) | 0.1002 (p=0.2155) | 0.1550 (p=0.1107) | 0.2846 (p=0.0113) | 0.3187 (p=0.0051) |
| 80 | -0.0263 (p=0.4184) | -0.0118 (p=0.4632) | 0.1139 (p=0.1852) | 0.0917 (p=0.2356) | 0.3784 (p=0.0010) |
| 81 | -0.0968 (p=0.2234) | 0.0354 (p=0.3907) | -0.0260 (p=0.4192) | 0.0789 (p=0.2677) | 0.4608 (p=0.0001) |
| 82 | -0.1610 (p=0.1019) | 0.1289 (p=0.1550) | 0.2327 (p=0.0321) | 0.2390 (p=0.0286) | 0.4097 (p=0.0004) |
| 83 | -0.1878 (p=0.0686) | 0.2184 (p=0.0415) | 0.1041 (p=0.2065) | 0.0111 (p=0.4653) | 0.2552 (p=0.0209) |
| 84 | 0.1351 (p=0.1435) | 0.0549 (p=0.3334) | 0.0773 (p=0.2718) | 0.0393 (p=0.3788) | 0.3783 (p=0.0010) |
| 85 | 0.1590 (p=0.1048) | -0.0056 (p=0.4824) | 0.0565 (p=0.3287) | 0.0759 (p=0.2754) | 0.5611 (p<0.0001) |
| 86 | -0.0406 (p=0.3752) | 0.1135 (p=0.1860) | 0.1787 (p=0.0788) | 0.1266 (p=0.1594) | 0.3313 (p=0.0037) |
| 87 | -0.1828 (p=0.0741) | -0.0063 (p=0.4802) | 0.1309 (p=0.1512) | 0.1341 (p=0.1453) | 0.4114 (p=0.0004) |
| 88 | 0.0131 (p=0.4592) | -0.0158 (p=0.4507) | 0.0665 (p=0.3007) | 0.0255 (p=0.4208) | 0.3813 (p=0.0009) |
| 89 | 0.0597 (p=0.3196) | 0.1529 (p=0.1139) | -0.0010 (p=0.4969) | 0.1301 (p=0.1527) | 0.4310 (p=0.0002) |
| 90 | -0.0470 (p=0.3562) | 0.0973 (p=0.2222) | 0.1097 (p=0.1941) | 0.0056 (p=0.4826) | 0.3815 (p=0.0009) |
| 91 | -0.0036 (p=0.4886) | 0.2690 (p=0.0158) | 0.1493 (p=0.1195) | 0.2011 (p=0.0555) | 0.3131 (p=0.0059) |
| 92 | -0.1468 (p=0.1235) | 0.2204 (p=0.0401) | 0.1716 (p=0.0876) | 0.2370 (p=0.0297) | 0.3938 (p=0.0006) |
| 93 | -0.1744 (p=0.0841) | 0.1145 (p=0.1838) | 0.1743 (p=0.0842) | 0.1124 (p=0.1882) | 0.3836 (p=0.0009) |
| 94 | 0.0046 (p=0.4855) | 0.2199 (p=0.0404) | 0.2741 (p=0.0142) | 0.2603 (p=0.0189) | 0.3592 (p=0.0018) |
| 95 | -0.0496 (p=0.3485) | 0.2028 (p=0.0540) | 0.1093 (p=0.1950) | -0.0086 (p=0.4731) | 0.4202 (p=0.0003) |
| 96 | -0.2034 (p=0.0535) | 0.0529 (p=0.3389) | 0.3850 (p=0.0008) | 0.2277 (p=0.0352) | 0.4509 (p=0.0001) |
| 97 | 0.0854 (p=0.2512) | 0.0100 (p=0.4689) | 0.1677 (p=0.0927) | 0.3096 (p=0.0064) | 0.3916 (p=0.0007) |
| 98 | -0.1070 (p=0.2001) | 0.2992 (p=0.0082) | 0.0938 (p=0.2306) | 0.0464 (p=0.3579) | 0.4481 (p=0.0001) |
| 99 | -0.1709 (p=0.0885) | 0.1772 (p=0.0806) | 0.1887 (p=0.0677) | 0.1665 (p=0.0943) | 0.3352 (p=0.0034) |
| 100 | -0.1199 (p=0.1727) | 0.2947 (p=0.0091) | 0.0728 (p=0.2839) | 0.0725 (p=0.2845) | 0.3303 (p=0.0038) |

**Supplementary Table 2b:** *Pearson correlation coefficient values and one-sided p-values for Monte Carlo data partitions for channel combinations without Framingham Risk Score.*

For every channel combination that does not include FRS, the Pearson correlation coefficient values and one-sided p-values were calculated. Channel combinations are indicated numerically with 1 = resting-state FC, 2 = cortical SA, 3 = cortical thickness, 4 = subcortical volume, 5 = FRS. MC = Monte Carlo, FC = functional connectivity, SA = surface area, FRS = Framingham Risk Score.

| **MC data partition** | **(1, 2)** | **(1, 3)** | **(1, 4)** | **(2, 3)** | **(2, 4)** | **(3, 4)** | **(1, 2, 3)** | **(1, 2, 4)** | **(1, 3, 4)** | **(2, 3, 4)** | **(1, 2, 3, 4)** |
| --- | --- | --- | --- | --- | --- | --- | --- | --- | --- | --- | --- |
| 1 | 0.0739 (p=0.2807) | 0.0549 (p=0.3334) | -0.1681 (p=0.9283) | 0.3277 (p=0.0041) | 0.1205 (p=0.1896) | 0.1192 (p=0.2080) | 0.2255 (p=0.0366) | -0.0304 (p=0.6409) | -0.0286 (p=0.6445) | 0.2132 (p=0.0586) | 0.1550 (p=0.1387) |
| 2 | 0.0553 (p=0.3321) | 0.1651 (p=0.0961) | 0.2290 (p=0.0424) | 0.1572 (p=0.1073) | 0.1745 (p=0.1016) | 0.1811 (p=0.0805) | 0.1652 (p=0.0961) | 0.1516 (p=0.1342) | 0.1821 (p=0.0793) | 0.1710 (p=0.0938) | 0.1744 (p=0.0897) |
| 3 | 0.0203 (p=0.4366) | 0.1293 (p=0.1542) | 0.0618 (p=0.3022) | 0.0272 (p=0.4154) | 0.0146 (p=0.4547) | 0.0811 (p=0.2627) | 0.0240 (p=0.4255) | 0.0188 (p=0.4416) | 0.1115 (p=0.1890) | 0.0284 (p=0.3726) | 0.0225 (p=0.4311) |
| 4 | -0.1035 (p=0.7923) | 0.0108 (p=0.4662) | 0.0823 (p=0.2134) | -0.0974 (p=0.7782) | -0.0994 (p=0.7775) | 0.0098 (p=0.4570) | -0.0976 (p=0.7786) | -0.0987 (p=0.7759) | 0.0295 (p=0.3916) | -0.0937 (p=0.7633) | -0.0939 (p=0.7636) |
| 5 | 0.0759 (p=0.2755) | 0.2338 (p=0.0315) | 0.0091 (p=0.5131) | 0.2605 (p=0.0188) | 0.0277 (p=0.4679) | 0.1409 (p=0.1927) | 0.2566 (p=0.0203) | 0.0154 (p=0.4986) | 0.1258 (p=0.2149) | 0.1375 (p=0.1952) | 0.1256 (p=0.2162) |
| 6 | -0.1808 (p=0.9236) | -0.1839 (p=0.9272) | -0.1370 (p=0.8630) | 0.0679 (p=0.2971) | 0.0743 (p=0.3686) | 0.0384 (p=0.4035) | -0.1739 (p=0.9154) | -0.0290 (p=0.6496) | -0.1229 (p=0.8312) | 0.1035 (p=0.2223) | -0.0879 (p=0.7588) |
| 7 | 0.1172 (p=0.1783) | 0.0059 (p=0.4815) | 0.0653 (p=0.3077) | 0.0218 (p=0.4320) | 0.2861 (p=0.0110) | 0.0193 (p=0.4377) | 0.0162 (p=0.4495) | 0.1956 (p=0.0615) | 0.0136 (p=0.4553) | 0.0306 (p=0.4030) | 0.0252 (p=0.4193) |
| 8 | 0.1104 (p=0.1926) | 0.0835 (p=0.2559) | 0.1006 (p=0.2025) | 0.0605 (p=0.3173) | 0.0894 (p=0.2277) | 0.0714 (p=0.2886) | 0.0702 (p=0.2908) | 0.1157 (p=0.1737) | 0.0787 (p=0.2695) | 0.0648 (p=0.3067) | 0.0696 (p=0.2936) |
| 9 | 0.0744 (p=0.2796) | -0.1792 (p=0.9218) | -0.0416 (p=0.5908) | 0.0883 (p=0.2438) | 0.1085 (p=0.1845) | 0.0812 (p=0.2479) | 0.0800 (p=0.2649) | 0.0369 (p=0.3529) | -0.0585 (p=0.6581) | 0.1035 (p=0.2369) | 0.0339 (p=0.3887) |
| 10 | -0.0700 (p=0.7086) | 0.0938 (p=0.2305) | -0.0941 (p=0.7282) | 0.0003 (p=0.4990) | 0.0019 (p=0.7711) | 0.0404 (p=0.3753) | 0.0466 (p=0.3572) | -0.1160 (p=0.8216) | 0.0665 (p=0.3009) | 0.0190 (p=0.4509) | 0.0341 (p=0.3979) |
| 11 | 0.2395 (p=0.0283) | 0.2426 (p=0.0267) | -0.0064 (p=0.2156) | 0.2432 (p=0.0264) | -0.0582 (p=0.3139) | -0.0808 (p=0.4682) | 0.2868 (p=0.0108) | 0.0765 (p=0.0766) | 0.0103 (p=0.1709) | 0.0388 (p=0.1110) | 0.0858 (p=0.0693) |
| 12 | -0.0558 (p=0.6691) | 0.1431 (p=0.1297) | 0.1791 (p=0.0368) | -0.0562 (p=0.6703) | -0.0494 (p=0.6496) | 0.1522 (p=0.0950) | -0.0558 (p=0.6691) | -0.0488 (p=0.6478) | 0.2228 (p=0.0357) | -0.0494 (p=0.6496) | -0.0488 (p=0.6478) |
| 13 | 0.0326 (p=0.3991) | -0.0411 (p=0.6265) | 0.1858 (p=0.0528) | -0.0223 (p=0.5693) | 0.0519 (p=0.3487) | -0.0340 (p=0.6051) | -0.0223 (p=0.5693) | 0.0416 (p=0.3732) | -0.0359 (p=0.6108) | -0.0195 (p=0.5607) | -0.0195 (p=0.5607) |
| 14 | -0.0862 (p=0.7509) | -0.0216 (p=0.5672) | -0.1744 (p=0.9154) | 0.2053 (p=0.0519) | -0.1864 (p=0.9317) | -0.0594 (p=0.6793) | -0.0268 (p=0.5831) | -0.1709 (p=0.9117) | -0.1496 (p=0.8800) | -0.0590 (p=0.6823) | -0.1449 (p=0.8737) |
| 15 | 0.1926 (p=0.0637) | 0.1991 (p=0.0573) | 0.0924 (p=0.2273) | 0.1417 (p=0.1320) | -0.0188 (p=0.5629) | 0.1269 (p=0.1590) | 0.1644 (p=0.0972) | 0.1131 (p=0.1900) | 0.1593 (p=0.1045) | 0.1325 (p=0.1485) | 0.1599 (p=0.1037) |
| 16 | 0.0156 (p=0.4514) | -0.0508 (p=0.6548) | -0.0039 (p=0.5259) | 0.2517 (p=0.0224) | 0.2503 (p=0.0387) | 0.1513 (p=0.1705) | 0.0247 (p=0.4231) | 0.1092 (p=0.2144) | -0.0002 (p=0.5396) | 0.2806 (p=0.0217) | 0.0414 (p=0.3866) |
| 17 | 0.2201 (p=0.0403) | 0.2912 (p=0.0098) | 0.1673 (p=0.0949) | 0.2083 (p=0.0493) | 0.0754 (p=0.2916) | 0.1797 (p=0.0746) | 0.2722 (p=0.0148) | 0.2001 (p=0.0587) | 0.2685 (p=0.0161) | 0.1808 (p=0.0806) | 0.2543 (p=0.0223) |
| 18 | 0.1333 (p=0.1469) | 0.1646 (p=0.0969) | 0.1043 (p=0.2557) | 0.1876 (p=0.0688) | 0.2462 (p=0.0912) | 0.0742 (p=0.4628) | 0.1897 (p=0.0667) | 0.1644 (p=0.1211) | 0.0304 (p=0.4319) | 0.1425 (p=0.1508) | 0.0554 (p=0.3517) |
| 19 | -0.0827 (p=0.7422) | 0.1386 (p=0.1373) | 0.0267 (p=0.3051) | 0.1383 (p=0.1378) | 0.0410 (p=0.2670) | 0.1486 (p=0.0793) | 0.1339 (p=0.1458) | 0.0297 (p=0.2862) | 0.1452 (p=0.0828) | 0.1511 (p=0.0767) | 0.1486 (p=0.0796) |
| 20 | 0.0816 (p=0.2608) | 0.0497 (p=0.3484) | 0.1355 (p=0.1370) | 0.0922 (p=0.2343) | 0.0959 (p=0.2221) | 0.1070 (p=0.2049) | 0.0932 (p=0.2319) | 0.0964 (p=0.2209) | 0.1020 (p=0.2127) | 0.1017 (p=0.2083) | 0.1023 (p=0.2070) |
| 21 | 0.1454 (p=0.1257) | 0.2425 (p=0.0267) | 0.1369 (p=0.1372) | 0.2390 (p=0.0286) | -0.0123 (p=0.5742) | 0.2334 (p=0.0303) | 0.2266 (p=0.0359) | 0.1395 (p=0.1411) | 0.2043 (p=0.0504) | 0.2361 (p=0.0279) | 0.1981 (p=0.0571) |
| 22 | 0.0781 (p=0.2698) | 0.1036 (p=0.2076) | -0.0347 (p=0.6060) | 0.0322 (p=0.4004) | 0.0564 (p=0.2865) | 0.0574 (p=0.3247) | 0.1259 (p=0.1608) | 0.0643 (p=0.2801) | 0.0855 (p=0.2535) | 0.0643 (p=0.3118) | 0.1149 (p=0.1733) |
| 23 | 0.0172 (p=0.4463) | 0.0899 (p=0.2399) | 0.0362 (p=0.3748) | 0.1860 (p=0.0706) | 0.1113 (p=0.1633) | 0.1297 (p=0.1269) | 0.1219 (p=0.1687) | 0.0059 (p=0.4580) | 0.0890 (p=0.2215) | 0.1488 (p=0.1365) | 0.1234 (p=0.1136) |
| 24 | -0.0263 (p=0.5816) | -0.0149 (p=0.5467) | -0.1151 (p=0.8755) | -0.0070 (p=0.5218) | -0.0405 (p=0.5387) | -0.1279 (p=0.8931) | -0.0275 (p=0.5855) | -0.0769 (p=0.7200) | -0.1019 (p=0.8725) | -0.0854 (p=0.6424) | -0.0928 (p=0.7824) |
| 25 | 0.0045 (p=0.4858) | 0.1936 (p=0.0627) | 0.1187 (p=0.1019) | 0.0327 (p=0.3988) | 0.0227 (p=0.4082) | 0.0168 (p=0.3701) | 0.0378 (p=0.3834) | 0.0239 (p=0.4035) | 0.0398 (p=0.3246) | 0.0469 (p=0.3483) | 0.0288 (p=0.3922) |
| 26 | 0.2265 (p=0.0357) | 0.1905 (p=0.0658) | 0.0392 (p=0.3207) | 0.1608 (p=0.1022) | 0.0954 (p=0.2254) | 0.1871 (p=0.0724) | 0.1711 (p=0.0883) | -0.0336 (p=0.5814) | 0.1893 (p=0.0682) | 0.1607 (p=0.1054) | 0.1628 (p=0.0977) |
| 27 | 0.1282 (p=0.1564) | 0.2462 (p=0.0249) | 0.0871 (p=0.2827) | 0.2418 (p=0.0271) | 0.1896 (p=0.0784) | 0.1730 (p=0.0638) | 0.2234 (p=0.0380) | 0.1594 (p=0.1370) | 0.1607 (p=0.0824) | 0.2095 (p=0.0915) | 0.2013 (p=0.0522) |
| 28 | 0.1288 (p=0.1552) | 0.1429 (p=0.1300) | -0.2008 (p=0.9472) | 0.1909 (p=0.0654) | -0.1079 (p=0.8085) | -0.0478 (p=0.6325) | 0.1623 (p=0.1001) | -0.1016 (p=0.7895) | -0.0477 (p=0.6221) | -0.0341 (p=0.6131) | -0.0405 (p=0.6209) |
| 29 | -0.0256 (p=0.5796) | 0.1166 (p=0.1795) | -0.1159 (p=0.7758) | 0.1053 (p=0.2037) | -0.0017 (p=0.5077) | 0.0740 (p=0.1543) | 0.0827 (p=0.2579) | -0.0396 (p=0.6207) | 0.1084 (p=0.1911) | 0.1520 (p=0.1102) | 0.0718 (p=0.2851) |
| 30 | 0.0492 (p=0.3496) | 0.0542 (p=0.3354) | -0.2057 (p=0.8630) | 0.0751 (p=0.2777) | -0.0698 (p=0.6381) | -0.0101 (p=0.4975) | 0.0748 (p=0.2784) | -0.0476 (p=0.6601) | -0.0026 (p=0.4728) | 0.0074 (p=0.4337) | 0.0500 (p=0.3443) |
| 31 | 0.0627 (p=0.3114) | -0.0616 (p=0.6857) | -0.2831 (p=0.9645) | 0.0914 (p=0.2363) | 0.1371 (p=0.0979) | 0.0286 (p=0.3732) | 0.0439 (p=0.3600) | 0.1502 (p=0.1540) | 0.0347 (p=0.3624) | 0.1342 (p=0.1278) | 0.1020 (p=0.1874) |
| 32 | 0.1447 (p=0.1271) | -0.0195 (p=0.5607) | -0.0169 (p=0.5371) | 0.2366 (p=0.0299) | 0.2410 (p=0.0251) | 0.2607 (p=0.0178) | 0.1507 (p=0.1172) | 0.1154 (p=0.1750) | -0.0046 (p=0.4982) | 0.2516 (p=0.0203) | 0.1196 (p=0.0796) |
| 33 | -0.2038 (p=0.9469) | 0.0513 (p=0.3435) | 0.0720 (p=0.5570) | -0.0869 (p=0.7526) | -0.0793 (p=0.7394) | 0.2084 (p=0.0740) | -0.1210 (p=0.8295) | -0.1003 (p=0.7854) | 0.1540 (p=0.2724) | -0.0392 (p=0.6224) | -0.0579 (p=0.6716) |
| 34 | 0.1314 (p=0.1504) | 0.2162 (p=0.0431) | 0.0811 (p=0.3026) | 0.2422 (p=0.0269) | 0.1478 (p=0.1353) | 0.2253 (p=0.0430) | 0.2401 (p=0.0280) | 0.1542 (p=0.1138) | 0.2273 (p=0.0412) | 0.1621 (p=0.1191) | 0.2438 (p=0.0294) |
| 35 | 0.0550 (p=0.3330) | 0.1149 (p=0.1830) | -0.0319 (p=0.6412) | 0.1211 (p=0.1703) | 0.0733 (p=0.2939) | 0.1099 (p=0.2049) | 0.1119 (p=0.1893) | 0.0621 (p=0.3222) | 0.1023 (p=0.2231) | 0.1135 (p=0.1909) | 0.1085 (p=0.2020) |
| 36 | -0.0589 (p=0.6780) | 0.0896 (p=0.2406) | 0.0397 (p=0.2672) | 0.0306 (p=0.4051) | -0.0546 (p=0.6501) | 0.1067 (p=0.2006) | 0.0329 (p=0.3981) | -0.0481 (p=0.6171) | 0.0979 (p=0.2160) | 0.0391 (p=0.3700) | 0.0320 (p=0.3870) |
| 37 | 0.2591 (p=0.0193) | 0.1008 (p=0.2141) | 0.2846 (p=0.0135) | 0.1969 (p=0.0595) | 0.3719 (p=0.0018) | 0.2307 (p=0.0369) | 0.2325 (p=0.0323) | 0.3830 (p=0.0013) | 0.2474 (p=0.0263) | 0.3118 (p=0.0076) | 0.2638 (p=0.0057) |
| 38 | -0.1463 (p=0.8756) | 0.0908 (p=0.2379) | -0.1060 (p=0.7576) | 0.0725 (p=0.2844) | -0.0298 (p=0.6041) | 0.1022 (p=0.1589) | 0.0596 (p=0.3201) | -0.0900 (p=0.7142) | 0.0781 (p=0.2941) | 0.0808 (p=0.2227) | 0.0299 (p=0.3371) |
| 39 | 0.2668 (p=0.0165) | 0.2597 (p=0.0191) | -0.2796 (p=0.9836) | 0.3286 (p=0.0040) | 0.2273 (p=0.0315) | 0.2152 (p=0.0311) | 0.3204 (p=0.0049) | 0.2149 (p=0.0443) | 0.2118 (p=0.0400) | 0.2925 (p=0.0078) | 0.2855 (p=0.0099) |
| 40 | 0.0894 (p=0.2411) | 0.1415 (p=0.1323) | 0.2104 (p=0.0632) | 0.1157 (p=0.1812) | 0.1067 (p=0.2105) | 0.2177 (p=0.0434) | 0.1064 (p=0.2014) | 0.1124 (p=0.1999) | 0.2326 (p=0.0352) | 0.1242 (p=0.1800) | 0.1257 (p=0.1758) |
| 41 | -0.0037 (p=0.5116) | 0.1592 (p=0.1045) | -0.0187 (p=0.5753) | 0.1372 (p=0.1399) | 0.1953 (p=0.1844) | 0.1141 (p=0.2817) | 0.1599 (p=0.1035) | -0.0035 (p=0.5281) | 0.1509 (p=0.1357) | 0.1549 (p=0.1812) | 0.1742 (p=0.1003) |
| 42 | 0.0476 (p=0.3544) | 0.0265 (p=0.4177) | 0.1229 (p=0.1236) | 0.0271 (p=0.4158) | 0.1014 (p=0.1969) | 0.1060 (p=0.1623) | 0.0261 (p=0.4189) | 0.1024 (p=0.1946) | 0.1139 (p=0.1410) | 0.0767 (p=0.2549) | 0.0767 (p=0.2549) |
| 43 | 0.2231 (p=0.0382) | 0.1286 (p=0.1556) | 0.0559 (p=0.3430) | 0.1302 (p=0.1526) | 0.0019 (p=0.5114) | 0.1084 (p=0.1977) | 0.1449 (p=0.1266) | 0.1130 (p=0.1951) | 0.1183 (p=0.1783) | 0.1214 (p=0.1700) | 0.1372 (p=0.1416) |
| 44 | 0.1221 (p=0.1682) | 0.0562 (p=0.3297) | 0.1011 (p=0.4128) | 0.0918 (p=0.2354) | 0.1203 (p=0.1630) | 0.0535 (p=0.3407) | 0.0858 (p=0.2501) | 0.0999 (p=0.1746) | 0.0481 (p=0.3565) | 0.0886 (p=0.2470) | 0.0841 (p=0.2586) |
| 45 | 0.0195 (p=0.4391) | 0.0968 (p=0.2233) | -0.0268 (p=0.5284) | 0.0846 (p=0.2531) | -0.0964 (p=0.7297) | 0.0138 (p=0.4123) | 0.0879 (p=0.2449) | -0.0375 (p=0.5624) | 0.0423 (p=0.3617) | -0.0003 (p=0.4531) | 0.0224 (p=0.3926) |
| 46 | 0.0477 (p=0.3542) | 0.2036 (p=0.0533) | -0.1844 (p=0.5438) | 0.2176 (p=0.0421) | 0.0197 (p=0.4867) | 0.1881 (p=0.0606) | 0.2300 (p=0.0338) | 0.0293 (p=0.4652) | 0.1922 (p=0.0562) | 0.2079 (p=0.0457) | 0.2150 (p=0.0405) |
| 47 | 0.1505 (p=0.1177) | -0.0141 (p=0.5439) | 0.1712 (p=0.0688) | 0.0171 (p=0.4467) | 0.2529 (p=0.0100) | 0.0470 (p=0.3575) | 0.0441 (p=0.3648) | 0.2484 (p=0.0115) | 0.0745 (p=0.2865) | 0.1009 (p=0.2254) | 0.1270 (p=0.1616) |
| 48 | 0.1772 (p=0.0807) | 0.0410 (p=0.3739) | 0.0550 (p=0.3468) | 0.1399 (p=0.1351) | 0.1750 (p=0.0824) | 0.0379 (p=0.3853) | 0.1051 (p=0.2043) | 0.1701 (p=0.0891) | 0.0379 (p=0.3853) | 0.1035 (p=0.2079) | 0.1002 (p=0.2154) |
| 49 | -0.0396 (p=0.6219) | 0.0402 (p=0.3763) | -0.0192 (p=0.5597) | 0.0602 (p=0.3184) | -0.0191 (p=0.5326) | 0.0442 (p=0.4099) | 0.0310 (p=0.4041) | -0.0443 (p=0.6811) | 0.0218 (p=0.4532) | 0.0318 (p=0.4248) | 0.0096 (p=0.4922) |
| 50 | 0.1640 (p=0.0977) | 0.0524 (p=0.3404) | 0.0400 (p=0.3939) | 0.1996 (p=0.0569) | 0.2126 (p=0.0488) | 0.2021 (p=0.0893) | 0.1783 (p=0.0793) | 0.1956 (p=0.0694) | 0.1123 (p=0.2214) | 0.2270 (p=0.0418) | 0.2052 (p=0.0607) |
| 51 | 0.0239 (p=0.4256) | 0.0315 (p=0.4024) | 0.1097 (p=0.2027) | 0.0466 (p=0.3576) | -0.0565 (p=0.6682) | 0.0969 (p=0.1442) | 0.0545 (p=0.3343) | 0.0100 (p=0.5795) | 0.0924 (p=0.2331) | -0.0096 (p=0.5502) | 0.0064 (p=0.5423) |
| 52 | 0.3247 (p=0.0044) | 0.0813 (p=0.2615) | 0.0819 (p=0.2897) | 0.0981 (p=0.2202) | 0.0810 (p=0.2780) | 0.0816 (p=0.2573) | 0.1056 (p=0.2032) | 0.0965 (p=0.2313) | 0.0841 (p=0.2508) | 0.0915 (p=0.2330) | 0.0895 (p=0.2377) |
| 53 | 0.2500 (p=0.0232) | 0.0953 (p=0.2270) | -0.0074 (p=0.1973) | 0.1447 (p=0.1270) | 0.0547 (p=0.3150) | 0.0290 (p=0.3415) | 0.1600 (p=0.1033) | 0.0464 (p=0.3333) | 0.0269 (p=0.3253) | 0.0695 (p=0.2439) | 0.0705 (p=0.2666) |
| 54 | 0.2256 (p=0.0366) | 0.0091 (p=0.4715) | 0.0657 (p=0.3463) | 0.0419 (p=0.3713) | 0.1660 (p=0.1622) | -0.0099 (p=0.5334) | 0.0290 (p=0.4099) | 0.1337 (p=0.2107) | -0.0033 (p=0.5245) | 0.0168 (p=0.4781) | 0.0141 (p=0.4999) |
| 55 | 0.2357 (p=0.0304) | 0.2140 (p=0.0447) | 0.1502 (p=0.1124) | 0.2187 (p=0.0413) | 0.2154 (p=0.0429) | 0.2126 (p=0.0456) | 0.2250 (p=0.0370) | 0.2510 (p=0.0220) | 0.2158 (p=0.0434) | 0.2215 (p=0.0390) | 0.2269 (p=0.0357) |
| 56 | 0.0990 (p=0.2182) | -0.0269 (p=0.5834) | 0.0354 (p=0.3668) | -0.0103 (p=0.5321) | 0.1071 (p=0.1940) | -0.0195 (p=0.5525) | -0.0189 (p=0.5591) | 0.0895 (p=0.1843) | -0.0145 (p=0.5385) | -0.0102 (p=0.5208) | -0.0160 (p=0.5385) |
| 57 | -0.0555 (p=0.6685) | 0.1763 (p=0.0818) | 0.1690 (p=0.0689) | -0.0148 (p=0.5462) | -0.0039 (p=0.4929) | 0.1184 (p=0.1555) | 0.0081 (p=0.4746) | -0.0078 (p=0.5035) | 0.1129 (p=0.1657) | 0.0618 (p=0.4068) | 0.0298 (p=0.4026) |
| 58 | -0.0050 (p=0.5155) | 0.1166 (p=0.1795) | -0.0869 (p=0.8227) | 0.1493 (p=0.1194) | -0.0277 (p=0.6571) | 0.1101 (p=0.3776) | 0.1256 (p=0.1614) | -0.0672 (p=0.7591) | 0.1176 (p=0.3653) | 0.1078 (p=0.4033) | 0.0622 (p=0.3641) |
| 59 | 0.0352 (p=0.3914) | 0.0081 (p=0.4747) | -0.0007 (p=0.7180) | 0.1269 (p=0.1589) | 0.1535 (p=0.1205) | 0.1026 (p=0.2260) | 0.0760 (p=0.2753) | 0.1024 (p=0.2411) | 0.0595 (p=0.3591) | 0.1557 (p=0.1150) | 0.1190 (p=0.1954) |
| 60 | -0.1067 (p=0.7994) | 0.1843 (p=0.0724) | -0.1869 (p=0.8910) | -0.0096 (p=0.5299) | -0.1237 (p=0.8256) | 0.1726 (p=0.0870) | -0.0368 (p=0.6136) | -0.1372 (p=0.8392) | 0.1164 (p=0.1553) | -0.0316 (p=0.6378) | -0.0541 (p=0.6537) |
| 61 | 0.0071 (p=0.4778) | 0.1918 (p=0.0645) | -0.0017 (p=0.6160) | 0.1429 (p=0.1300) | 0.0896 (p=0.2597) | 0.2254 (p=0.0754) | 0.1299 (p=0.1531) | 0.0645 (p=0.3131) | 0.2175 (p=0.2424) | 0.0361 (p=0.3834) | -0.0373 (p=0.6459) |
| 62 | 0.0500 (p=0.3473) | 0.1270 (p=0.1587) | 0.1878 (p=0.0680) | 0.0396 (p=0.3780) | 0.0511 (p=0.3432) | 0.1332 (p=0.1517) | 0.0696 (p=0.2924) | 0.0747 (p=0.2821) | 0.1627 (p=0.1032) | 0.0580 (p=0.3233) | 0.0847 (p=0.2561) |
| 63 | 0.0252 (p=0.4218) | 0.1408 (p=0.1336) | -0.1254 (p=0.8312) | 0.2014 (p=0.0552) | -0.0016 (p=0.4384) | 0.0826 (p=0.2139) | 0.1797 (p=0.0776) | -0.0243 (p=0.4932) | 0.0679 (p=0.2338) | 0.1130 (p=0.1485) | 0.0889 (p=0.1661) |
| 64 | -0.0221 (p=0.5688) | 0.0820 (p=0.2597) | 0.2176 (p=0.0415) | -0.0018 (p=0.5055) | 0.0072 (p=0.4574) | 0.1239 (p=0.1471) | -0.0160 (p=0.5500) | 0.0072 (p=0.4574) | 0.1805 (p=0.1376) | 0.0539 (p=0.3338) | 0.0397 (p=0.3735) |
| 65 | -0.0201 (p=0.5628) | 0.0836 (p=0.2558) | 0.0781 (p=0.2811) | 0.0750 (p=0.2780) | -0.0316 (p=0.4455) | 0.1003 (p=0.2198) | 0.0675 (p=0.2981) | -0.0384 (p=0.4823) | 0.0926 (p=0.2368) | 0.1024 (p=0.2778) | 0.0874 (p=0.3099) |
| 66 | 0.0709 (p=0.2888) | 0.1324 (p=0.1485) | 0.1582 (p=0.1082) | 0.3371 (p=0.0032) | 0.1444 (p=0.1527) | 0.1918 (p=0.0925) | 0.1753 (p=0.0830) | 0.1637 (p=0.2245) | 0.1928 (p=0.0662) | 0.2070 (p=0.0730) | 0.1889 (p=0.0689) |
| 67 | -0.0541 (p=0.6645) | -0.1226 (p=0.8327) | -0.0269 (p=0.5802) | -0.0240 (p=0.5747) | -0.0475 (p=0.6540) | -0.0350 (p=0.6085) | -0.0328 (p=0.6016) | -0.0508 (p=0.6551) | -0.0485 (p=0.6482) | -0.0235 (p=0.5742) | -0.0305 (p=0.5945) |
| 68 | 0.0020 (p=0.4937) | 0.0853 (p=0.2514) | 0.1818 (p=0.1290) | 0.0376 (p=0.3841) | 0.0020 (p=0.5466) | 0.0740 (p=0.2980) | 0.0684 (p=0.2957) | 0.0469 (p=0.4071) | 0.1036 (p=0.2250) | 0.0612 (p=0.3425) | 0.0925 (p=0.2672) |
| 69 | 0.1568 (p=0.1080) | 0.2116 (p=0.0466) | -0.0705 (p=0.7272) | 0.2463 (p=0.0249) | -0.0279 (p=0.6093) | -0.0397 (p=0.6326) | 0.2247 (p=0.0371) | -0.0331 (p=0.6258) | -0.0447 (p=0.6480) | -0.0047 (p=0.5280) | -0.0099 (p=0.5449) |
| 70 | -0.0498 (p=0.6521) | -0.0347 (p=0.6071) | 0.1748 (p=0.0861) | 0.0456 (p=0.3601) | 0.3892 (p=0.0008) | 0.0950 (p=0.2239) | -0.0443 (p=0.6360) | 0.2331 (p=0.0285) | 0.0789 (p=0.2640) | 0.1427 (p=0.1081) | 0.0606 (p=0.2899) |
| 71 | -0.0121 (p=0.5378) | 0.0022 (p=0.4932) | 0.1042 (p=0.1807) | -0.0247 (p=0.5769) | 0.0303 (p=0.3977) | 0.0975 (p=0.2088) | -0.0139 (p=0.5434) | 0.0329 (p=0.3889) | 0.1070 (p=0.1726) | 0.0322 (p=0.3915) | 0.0384 (p=0.3723) |
| 72 | -0.1960 (p=0.9397) | 0.0478 (p=0.3538) | 0.1715 (p=0.0639) | 0.0009 (p=0.4971) | -0.0483 (p=0.5817) | 0.2254 (p=0.0427) | -0.0162 (p=0.5506) | -0.0721 (p=0.6469) | 0.1999 (p=0.0743) | 0.0765 (p=0.3377) | 0.0568 (p=0.3704) |
| 73 | -0.0114 (p=0.5355) | 0.1157 (p=0.1813) | -0.0541 (p=0.6047) | 0.1842 (p=0.0726) | 0.2828 (p=0.0057) | 0.1700 (p=0.0844) | 0.1382 (p=0.1380) | 0.0360 (p=0.3214) | 0.1286 (p=0.1468) | 0.1886 (p=0.0628) | 0.1481 (p=0.1128) |
| 74 | 0.0423 (p=0.3699) | 0.2612 (p=0.0185) | 0.2194 (p=0.0281) | 0.0315 (p=0.4025) | 0.0260 (p=0.4205) | 0.1510 (p=0.1035) | 0.0433 (p=0.3670) | 0.0377 (p=0.3848) | 0.2598 (p=0.0164) | 0.0294 (p=0.4123) | 0.0393 (p=0.3820) |
| 75 | 0.0560 (p=0.3301) | 0.0581 (p=0.3243) | -0.0896 (p=0.7505) | 0.0545 (p=0.3316) | 0.1483 (p=0.1004) | 0.1248 (p=0.1614) | 0.0665 (p=0.3007) | 0.0516 (p=0.3176) | 0.0618 (p=0.3069) | 0.1797 (p=0.0688) | 0.0792 (p=0.2552) |
| 76 | 0.0523 (p=0.3406) | 0.0867 (p=0.2479) | -0.0279 (p=0.6209) | 0.1128 (p=0.1875) | 0.0771 (p=0.2718) | 0.0944 (p=0.2242) | 0.1017 (p=0.2120) | 0.0606 (p=0.3160) | 0.0860 (p=0.2440) | 0.1145 (p=0.1839) | 0.1073 (p=0.1994) |
| 77 | -0.0971 (p=0.7774) | -0.0410 (p=0.6261) | -0.1701 (p=0.8675) | 0.0785 (p=0.2687) | -0.0014 (p=0.4535) | 0.1252 (p=0.1253) | -0.0457 (p=0.6401) | -0.0921 (p=0.7091) | -0.0750 (p=0.6499) | 0.0807 (p=0.2299) | 0.0191 (p=0.5908) |
| 78 | 0.0760 (p=0.2752) | 0.0974 (p=0.2219) | 0.0424 (p=0.3629) | 0.1248 (p=0.1629) | 0.1329 (p=0.1566) | 0.1066 (p=0.2043) | 0.1233 (p=0.1658) | 0.0987 (p=0.2167) | 0.1042 (p=0.2082) | 0.1215 (p=0.1721) | 0.1210 (p=0.1716) |
| 79 | 0.1938 (p=0.0624) | 0.1822 (p=0.0748) | 0.0407 (p=0.3988) | 0.2775 (p=0.0132) | 0.1171 (p=0.1942) | 0.0985 (p=0.2357) | 0.2790 (p=0.0128) | 0.1157 (p=0.1926) | 0.0765 (p=0.2862) | 0.1530 (p=0.1212) | 0.1431 (p=0.1338) |
| 80 | 0.1991 (p=0.0577) | 0.0801 (p=0.2646) | 0.0572 (p=0.3192) | 0.3026 (p=0.0075) | 0.2996 (p=0.0077) | 0.1162 (p=0.1703) | 0.2998 (p=0.0080) | 0.2839 (p=0.0088) | 0.1100 (p=0.1778) | 0.3034 (p=0.0070) | 0.2973 (p=0.0081) |
| 81 | 0.1219 (p=0.1685) | 0.1218 (p=0.1689) | 0.0557 (p=0.3198) | 0.1674 (p=0.0931) | 0.0949 (p=0.2130) | 0.0846 (p=0.1703) | 0.1668 (p=0.0938) | 0.0748 (p=0.2284) | 0.0672 (p=0.2674) | 0.1348 (p=0.1228) | 0.1205 (p=0.1620) |
| 82 | 0.0496 (p=0.3486) | 0.0792 (p=0.2668) | 0.0761 (p=0.2801) | 0.2542 (p=0.0213) | 0.1229 (p=0.1292) | 0.1446 (p=0.0997) | 0.0927 (p=0.2333) | 0.1012 (p=0.2139) | 0.0961 (p=0.2282) | 0.1589 (p=0.0717) | 0.1195 (p=0.1691) |
| 83 | 0.1397 (p=0.1355) | 0.1338 (p=0.1459) | 0.0227 (p=0.5388) | 0.1813 (p=0.0758) | 0.2067 (p=0.0406) | 0.1135 (p=0.1865) | 0.1631 (p=0.0989) | 0.1212 (p=0.1470) | 0.1065 (p=0.2195) | 0.1750 (p=0.0901) | 0.1444 (p=0.1375) |
| 84 | -0.0692 (p=0.7065) | -0.0745 (p=0.7208) | -0.0924 (p=0.7605) | 0.0798 (p=0.2654) | 0.1017 (p=0.1688) | 0.0237 (p=0.4105) | -0.0626 (p=0.6885) | -0.0724 (p=0.7094) | -0.0855 (p=0.7408) | 0.0582 (p=0.3139) | -0.0651 (p=0.6864) |
| 85 | -0.0569 (p=0.6725) | 0.1034 (p=0.2081) | 0.0224 (p=0.5523) | 0.1044 (p=0.2058) | -0.0021 (p=0.5015) | 0.1013 (p=0.2129) | 0.1090 (p=0.1957) | 0.0221 (p=0.4262) | 0.1037 (p=0.2074) | 0.1240 (p=0.1646) | 0.1273 (p=0.1583) |
| 86 | 0.1483 (p=0.1211) | 0.1582 (p=0.1059) | 0.0658 (p=0.2909) | 0.2266 (p=0.0359) | 0.1354 (p=0.1437) | 0.1595 (p=0.1056) | 0.2217 (p=0.0392) | 0.1453 (p=0.1226) | 0.1598 (p=0.1020) | 0.2022 (p=0.0685) | 0.1859 (p=0.0695) |
| 87 | 0.0665 (p=0.3009) | 0.1244 (p=0.1637) | 0.1525 (p=0.1170) | 0.1475 (p=0.1223) | 0.1002 (p=0.2094) | 0.1477 (p=0.1235) | 0.1408 (p=0.1335) | 0.1114 (p=0.1889) | 0.1586 (p=0.1058) | 0.1527 (p=0.1208) | 0.1619 (p=0.0998) |
| 88 | 0.0405 (p=0.3752) | 0.1292 (p=0.1545) | -0.0354 (p=0.6281) | 0.1343 (p=0.1450) | 0.0015 (p=0.5191) | 0.0159 (p=0.4640) | 0.1224 (p=0.1676) | -0.0047 (p=0.5372) | 0.0070 (p=0.4963) | 0.0420 (p=0.3966) | 0.0334 (p=0.4258) |
| 89 | -0.0083 (p=0.5261) | -0.0085 (p=0.5264) | -0.0455 (p=0.6501) | 0.0286 (p=0.4111) | 0.1080 (p=0.2294) | 0.0304 (p=0.4102) | 0.0044 (p=0.4863) | 0.0189 (p=0.4632) | 0.0007 (p=0.4991) | 0.0423 (p=0.3713) | 0.0129 (p=0.4582) |
| 90 | 0.2570 (p=0.0202) | 0.0527 (p=0.3397) | 0.0192 (p=0.4516) | 0.0712 (p=0.2881) | 0.0211 (p=0.4458) | 0.0194 (p=0.4528) | 0.0755 (p=0.2765) | 0.0211 (p=0.4458) | 0.0194 (p=0.4528) | 0.0214 (p=0.4469) | 0.0214 (p=0.4469) |
| 91 | 0.2945 (p=0.0091) | 0.2264 (p=0.0360) | 0.0303 (p=0.6427) | 0.2502 (p=0.0231) | 0.3001 (p=0.0086) | 0.2265 (p=0.0419) | 0.3066 (p=0.0069) | 0.3100 (p=0.0378) | 0.2294 (p=0.0354) | 0.2534 (p=0.0211) | 0.3101 (p=0.0062) |
| 92 | 0.0516 (p=0.3429) | 0.0663 (p=0.3013) | -0.0359 (p=0.6733) | 0.1499 (p=0.1186) | 0.1244 (p=0.1753) | 0.1100 (p=0.2059) | 0.1151 (p=0.1825) | 0.0737 (p=0.3048) | 0.0722 (p=0.3082) | 0.1339 (p=0.1536) | 0.1064 (p=0.2137) |
| 93 | 0.2447 (p=0.0257) | 0.1855 (p=0.0711) | 0.0259 (p=0.3118) | 0.3742 (p=0.0012) | 0.2203 (p=0.0302) | 0.1428 (p=0.0877) | 0.3452 (p=0.0026) | 0.3067 (p=0.0062) | 0.1700 (p=0.0897) | 0.2652 (p=0.0094) | 0.2723 (p=0.0145) |
| 94 | -0.0485 (p=0.6483) | -0.1035 (p=0.7921) | -0.0358 (p=0.5155) | 0.0735 (p=0.2820) | 0.0103 (p=0.4677) | 0.1325 (p=0.2000) | -0.0398 (p=0.6225) | -0.0229 (p=0.5728) | 0.0253 (p=0.4078) | 0.0217 (p=0.4439) | -0.0112 (p=0.5281) |
| 95 | -0.0153 (p=0.5477) | 0.0508 (p=0.3450) | -0.0213 (p=0.5359) | 0.0882 (p=0.2442) | -0.0285 (p=0.5911) | -0.0029 (p=0.4697) | 0.0716 (p=0.2869) | -0.0331 (p=0.6340) | 0.0132 (p=0.4176) | 0.0163 (p=0.4926) | 0.0074 (p=0.5220) |
| 96 | -0.1248 (p=0.8371) | -0.1768 (p=0.9189) | 0.0055 (p=0.4964) | -0.1848 (p=0.9281) | 0.0397 (p=0.4043) | 0.0338 (p=0.4332) | -0.1430 (p=0.8702) | 0.0136 (p=0.4797) | 0.0049 (p=0.5181) | 0.0355 (p=0.4304) | 0.0111 (p=0.5018) |
| 97 | -0.0305 (p=0.5991) | 0.1468 (p=0.1235) | -0.0623 (p=0.7064) | 0.1961 (p=0.0602) | 0.0719 (p=0.2901) | 0.2145 (p=0.0420) | 0.1047 (p=0.2032) | 0.0276 (p=0.4149) | 0.0924 (p=0.2205) | 0.1602 (p=0.0978) | 0.0965 (p=0.2129) |
| 98 | -0.0315 (p=0.5976) | 0.0158 (p=0.4508) | 0.0181 (p=0.4549) | -0.0560 (p=0.6697) | -0.0773 (p=0.6704) | 0.0141 (p=0.4697) | -0.0295 (p=0.5916) | -0.0598 (p=0.6484) | 0.0126 (p=0.4718) | -0.0682 (p=0.6607) | -0.0652 (p=0.6573) |
| 99 | 0.2238 (p=0.0377) | 0.2615 (p=0.0184) | 0.0269 (p=0.3194) | 0.2915 (p=0.0097) | 0.2258 (p=0.0359) | 0.2655 (p=0.0156) | 0.2885 (p=0.0104) | 0.2238 (p=0.0369) | 0.2626 (p=0.0162) | 0.2861 (p=0.0107) | 0.2845 (p=0.0109) |
| 100 | 0.1477 (p=0.1221) | 0.1191 (p=0.1744) | 0.0506 (p=0.3408) | 0.1501 (p=0.1183) | 0.1663 (p=0.0968) | 0.1501 (p=0.1181) | 0.1797 (p=0.0777) | 0.1628 (p=0.1007) | 0.1314 (p=0.1457) | 0.1534 (p=0.1103) | 0.1874 (p=0.0683) |

**Supplementary Table 2c:** *Pearson correlation coefficient values and one-sided p-values for Monte Carlo data partitions for channel combinations including Framingham Risk Score.*

For every channel combination that includes FRS, the Pearson correlation coefficient values and one-sided p-values were calculated. Channel combinations are indicated numerically with 1 = resting-state FC, 2 = cortical SA, 3 = cortical thickness, 4 = subcortical volume, 5 = FRS. MC = Monte Carlo, FC = functional connectivity, SA = surface area, FRS = Framingham Risk Score.

| **MC data partition** | **(1, 5)** | **(2, 5)** | **(3, 5)** | **(4, 5)** | **(1, 2, 5)** | **(1, 3, 5)** | **(1, 4, 5)** | **(2, 3, 5)** | **(2, 4, 5)** | **(3, 4, 5)** | **(1, 2, 3, 5)** | **(1, 2, 4, 5)** | **(1, 3, 4, 5)** | **(2, 3, 4, 5)** | **(1, 2, 3,**  **4, 5)** |
| --- | --- | --- | --- | --- | --- | --- | --- | --- | --- | --- | --- | --- | --- | --- | --- |
| 1 | 0.3361 (p=0.0033) | 0.4103 (p=0.0004) | 0.3405 (p=0.0030) | 0.3438 (p=0.0023) | 0.3311 (p=0.0038) | 0.3271 (p=0.0042) | 0.2889 (p=0.0088) | 0.4651 (p=0.0001) | 0.3844 (p=0.0008) | 0.3417 (p=0.0025) | 0.3692 (p=0.0013) | 0.3323 (p=0.0032) | 0.2963 (p=0.0076) | 0.3845 (p=0.0008) | 0.3389 (p=0.0027) |
| 2 | 0.3684 (p=0.0014) | 0.3680 (p=0.0014) | 0.4148 (p=0.0003) | 0.3684 (p=0.0014) | 0.3680 (p=0.0014) | 0.3941 (p=0.0006) | 0.3684 (p=0.0014) | 0.3693 (p=0.0013) | 0.3680 (p=0.0014) | 0.3697 (p=0.0013) | 0.3693 (p=0.0013) | 0.3680 (p=0.0014) | 0.3697 (p=0.0013) | 0.3693 (p=0.0013) | 0.3693 (p=0.0013) |
| 3 | 0.5543 (p<0.0001) | 0.4206 (p=0.0003) | 0.5107 (p<0.0001) | 0.5480 (p<0.0001) | 0.4340 (p=0.0002) | 0.5547 (p<0.0001) | 0.5544 (p<0.0001) | 0.4356 (p=0.0002) | 0.4335 (p=0.0002) | 0.5531 (p<0.0001) | 0.4436 (p=0.0001) | 0.4454 (p=0.0001) | 0.5548 (p<0.0001) | 0.4453 (p=0.0001) | 0.4545 (p=0.0001) |
| 4 | 0.2168 (p=0.0426) | 0.1516 (p=0.1159) | 0.2168 (p=0.0426) | 0.2168 (p=0.0426) | 0.1530 (p=0.1137) | 0.2168 (p=0.0426) | 0.2168 (p=0.0426) | 0.1469 (p=0.1234) | 0.1568 (p=0.1070) | 0.2168 (p=0.0426) | 0.1484 (p=0.1209) | 0.1598 (p=0.1022) | 0.2168 (p=0.0426) | 0.1526 (p=0.1132) | 0.1540 (p=0.1110) |
| 5 | 0.4114 (p=0.0004) | 0.4190 (p=0.0003) | 0.3651 (p=0.0015) | 0.3595 (p=0.0022) | 0.4441 (p=0.0001) | 0.4037 (p=0.0005) | 0.3496 (p=0.0019) | 0.4199 (p=0.0003) | 0.3888 (p=0.0010) | 0.3739 (p=0.0014) | 0.4496 (p=0.0001) | 0.3873 (p=0.0011) | 0.4067 (p=0.0016) | 0.4002 (p=0.0010) | 0.4090 (p=0.0005) |
| 6 | 0.2450 (p=0.0255) | 0.3523 (p=0.0022) | 0.3529 (p=0.0021) | 0.3230 (p=0.0043) | 0.2611 (p=0.0186) | 0.2638 (p=0.0176) | 0.2558 (p=0.0195) | 0.3424 (p=0.0028) | 0.3137 (p=0.0068) | 0.3231 (p=0.0044) | 0.2734 (p=0.0144) | 0.2597 (p=0.0175) | 0.2677 (p=0.0156) | 0.3272 (p=0.0040) | 0.2771 (p=0.0127) |
| 7 | 0.3056 (p=0.0070) | 0.3448 (p=0.0026) | 0.2814 (p=0.0122) | 0.3408 (p=0.0029) | 0.3448 (p=0.0026) | 0.1807 (p=0.0765) | 0.3220 (p=0.0045) | 0.3269 (p=0.0042) | 0.3448 (p=0.0026) | 0.3167 (p=0.0051) | 0.3051 (p=0.0071) | 0.3752 (p=0.0011) | 0.2842 (p=0.0114) | 0.3390 (p=0.0031) | 0.3159 (p=0.0055) |
| 8 | 0.3890 (p=0.0007) | 0.4360 (p=0.0002) | 0.3940 (p=0.0006) | 0.4033 (p=0.0005) | 0.3962 (p=0.0006) | 0.3940 (p=0.0006) | 0.3940 (p=0.0006) | 0.3962 (p=0.0006) | 0.4381 (p=0.0001) | 0.3940 (p=0.0006) | 0.3962 (p=0.0006) | 0.3962 (p=0.0006) | 0.3940 (p=0.0006) | 0.3962 (p=0.0006) | 0.3962 (p=0.0006) |
| 9 | 0.3302 (p=0.0038) | 0.2827 (p=0.0118) | 0.3295 (p=0.0039) | 0.2975 (p=0.0077) | 0.2809 (p=0.0123) | 0.3295 (p=0.0039) | 0.2970 (p=0.0079) | 0.2919 (p=0.0096) | 0.2572 (p=0.0158) | 0.2957 (p=0.0083) | 0.2778 (p=0.0129) | 0.2505 (p=0.0149) | 0.2966 (p=0.0082) | 0.2714 (p=0.0140) | 0.2583 (p=0.0149) |
| 10 | 0.5139 (p<0.0001) | 0.5047 (p<0.0001) | 0.4780 (p<0.0001) | 0.4784 (p<0.0001) | 0.5047 (p<0.0001) | 0.4359 (p=0.0002) | 0.4754 (p<0.0001) | 0.4919 (p<0.0001) | 0.5025 (p<0.0001) | 0.3579 (p=0.0019) | 0.5060 (p<0.0001) | 0.5025 (p<0.0001) | 0.4802 (p<0.0001) | 0.4937 (p<0.0001) | 0.5030 (p<0.0001) |
| 11 | 0.3127 (p=0.0059) | 0.3746 (p=0.0011) | 0.3127 (p=0.0059) | 0.3127 (p=0.0059) | 0.3127 (p=0.0059) | 0.3127 (p=0.0059) | 0.3127 (p=0.0059) | 0.3549 (p=0.0020) | 0.3127 (p=0.0059) | 0.3127 (p=0.0059) | 0.3127 (p=0.0059) | 0.3127 (p=0.0059) | 0.3127 (p=0.0059) | 0.3127 (p=0.0059) | 0.3127 (p=0.0059) |
| 12 | 0.4060 (p=0.0004) | 0.2645 (p=0.0174) | 0.4060 (p=0.0004) | 0.4060 (p=0.0004) | 0.2674 (p=0.0163) | 0.4060 (p=0.0004) | 0.4060 (p=0.0004) | 0.2677 (p=0.0162) | 0.2705 (p=0.0157) | 0.4060 (p=0.0004) | 0.2698 (p=0.0155) | 0.2726 (p=0.0150) | 0.4060 (p=0.0004) | 0.2721 (p=0.0153) | 0.2745 (p=0.0145) |
| 13 | 0.2701 (p=0.0154) | 0.2451 (p=0.0254) | 0.1805 (p=0.0768) | 0.2701 (p=0.0154) | 0.2376 (p=0.0293) | 0.1812 (p=0.0760) | 0.2701 (p=0.0154) | 0.1991 (p=0.0574) | 0.2402 (p=0.0282) | 0.1879 (p=0.0691) | 0.1941 (p=0.0622) | 0.2345 (p=0.0314) | 0.1888 (p=0.0681) | 0.1955 (p=0.0607) | 0.1953 (p=0.0609) |
| 14 | 0.4646 (p=0.0001) | 0.4688 (p<0.0001) | 0.4682 (p<0.0001) | 0.4538 (p=0.0001) | 0.4663 (p=0.0001) | 0.4657 (p=0.0001) | 0.4510 (p=0.0001) | 0.4700 (p<0.0001) | 0.4554 (p=0.0001) | 0.4543 (p=0.0001) | 0.4675 (p<0.0001) | 0.4527 (p=0.0001) | 0.4515 (p=0.0001) | 0.4561 (p=0.0001) | 0.4532 (p=0.0001) |
| 15 | 0.3825 (p=0.0009) | 0.3825 (p=0.0009) | 0.3917 (p=0.0007) | 0.3825 (p=0.0009) | 0.3825 (p=0.0009) | 0.4650 (p=0.0007) | 0.3825 (p=0.0009) | 0.3917 (p=0.0007) | 0.3825 (p=0.0009) | 0.3482 (p=0.0039) | 0.4336 (p=0.0002) | 0.3825 (p=0.0009) | 0.4098 (p=0.0009) | 0.3377 (p=0.0047) | 0.3577 (p=0.0029) |
| 16 | 0.1669 (p=0.0937) | 0.3071 (p=0.0068) | 0.1915 (p=0.0648) | 0.1915 (p=0.0648) | 0.2900 (p=0.0100) | 0.1915 (p=0.0648) | 0.1796 (p=0.0784) | 0.1915 (p=0.0648) | 0.1915 (p=0.0648) | 0.1915 (p=0.0648) | 0.2522 (p=0.0222) | 0.2903 (p=0.0108) | 0.1915 (p=0.0648) | 0.1915 (p=0.0648) | 0.2572 (p=0.0201) |
| 17 | 0.4048 (p=0.0005) | 0.4061 (p=0.0004) | 0.4025 (p=0.0005) | 0.4015 (p=0.0005) | 0.4086 (p=0.0004) | 0.4058 (p=0.0004) | 0.4048 (p=0.0005) | 0.4065 (p=0.0004) | 0.4061 (p=0.0004) | 0.4025 (p=0.0005) | 0.4090 (p=0.0004) | 0.4086 (p=0.0004) | 0.4058 (p=0.0004) | 0.4065 (p=0.0004) | 0.4090 (p=0.0004) |
| 18 | 0.3119 (p=0.0061) | 0.3317 (p=0.0037) | 0.2902 (p=0.0100) | 0.3458 (p=0.0022) | 0.3124 (p=0.0060) | 0.3144 (p=0.0057) | 0.3140 (p=0.0058) | 0.3255 (p=0.0043) | 0.3526 (p=0.0020) | 0.2697 (p=0.0136) | 0.3150 (p=0.0056) | 0.3146 (p=0.0057) | 0.2834 (p=0.0104) | 0.3179 (p=0.0052) | 0.3100 (p=0.0041) |
| 19 | 0.2982 (p=0.0083) | 0.2909 (p=0.0098) | 0.2849 (p=0.0113) | 0.3113 (p=0.0060) | 0.2897 (p=0.0101) | 0.2961 (p=0.0088) | 0.2817 (p=0.0131) | 0.2949 (p=0.0090) | 0.2817 (p=0.0131) | 0.2812 (p=0.0089) | 0.2988 (p=0.0082) | 0.2817 (p=0.0131) | 0.2812 (p=0.0132) | 0.3178 (p=0.0052) | 0.3222 (p=0.0048) |
| 20 | 0.5219 (p<0.0001) | 0.4881 (p<0.0001) | 0.5178 (p<0.0001) | 0.5219 (p<0.0001) | 0.4870 (p<0.0001) | 0.5178 (p<0.0001) | 0.5219 (p<0.0001) | 0.4852 (p<0.0001) | 0.4870 (p<0.0001) | 0.5178 (p<0.0001) | 0.4853 (p<0.0001) | 0.4848 (p<0.0001) | 0.5178 (p<0.0001) | 0.4809 (p<0.0001) | 0.4823 (p<0.0001) |
| 21 | 0.3816 (p=0.0009) | 0.3816 (p=0.0009) | 0.3816 (p=0.0009) | 0.3816 (p=0.0009) | 0.3816 (p=0.0009) | 0.3816 (p=0.0009) | 0.3816 (p=0.0009) | 0.3816 (p=0.0009) | 0.3816 (p=0.0009) | 0.3816 (p=0.0009) | 0.3816 (p=0.0009) | 0.3816 (p=0.0009) | 0.3816 (p=0.0009) | 0.3816 (p=0.0009) | 0.3816 (p=0.0009) |
| 22 | 0.4624 (p=0.0001) | 0.4325 (p=0.0002) | 0.4624 (p=0.0001) | 0.3997 (p=0.0006) | 0.4326 (p=0.0002) | 0.4624 (p=0.0001) | 0.4624 (p=0.0001) | 0.4314 (p=0.0002) | 0.4319 (p=0.0002) | 0.4042 (p=0.0001) | 0.3748 (p=0.0011) | 0.3899 (p=0.0008) | 0.4624 (p=0.0001) | 0.4328 (p=0.0002) | 0.3738 (p=0.0013) |
| 23 | 0.3606 (p=0.0017) | 0.3869 (p=0.0008) | 0.3760 (p=0.0011) | 0.3760 (p=0.0011) | 0.3741 (p=0.0012) | 0.3737 (p=0.0012) | 0.3737 (p=0.0012) | 0.3719 (p=0.0012) | 0.3767 (p=0.0011) | 0.3760 (p=0.0011) | 0.3741 (p=0.0012) | 0.3741 (p=0.0012) | 0.3737 (p=0.0012) | 0.3767 (p=0.0011) | 0.3741 (p=0.0012) |
| 24 | 0.2588 (p=0.0195) | 0.2314 (p=0.0329) | 0.2542 (p=0.0214) | 0.2168 (p=0.0314) | 0.2563 (p=0.0205) | 0.2096 (p=0.0482) | 0.1438 (p=0.0788) | 0.2426 (p=0.0267) | 0.2127 (p=0.0508) | 0.1930 (p=0.0629) | 0.2191 (p=0.0409) | 0.1970 (p=0.0584) | 0.1271 (p=0.1547) | 0.2049 (p=0.0521) | 0.1712 (p=0.0869) |
| 25 | 0.4555 (p=0.0001) | 0.3765 (p=0.0011) | 0.4555 (p=0.0001) | 0.4549 (p=0.0001) | 0.3985 (p=0.0006) | 0.4744 (p<0.0001) | 0.4549 (p=0.0001) | 0.4131 (p=0.0003) | 0.4491 (p=0.0001) | 0.4549 (p=0.0001) | 0.4260 (p=0.0002) | 0.4041 (p=0.0004) | 0.4549 (p=0.0001) | 0.4491 (p=0.0001) | 0.4225 (p=0.0002) |
| 26 | 0.2347 (p=0.0310) | 0.2009 (p=0.0557) | 0.1593 (p=0.1043) | 0.2347 (p=0.0310) | 0.2073 (p=0.0501) | 0.1531 (p=0.1136) | 0.2347 (p=0.0310) | 0.1285 (p=0.1559) | 0.1969 (p=0.0598) | 0.1519 (p=0.1155) | 0.1340 (p=0.1456) | 0.2039 (p=0.0539) | 0.1527 (p=0.1161) | 0.0968 (p=0.1716) | 0.1347 (p=0.1455) |
| 27 | 0.3426 (p=0.0028) | 0.3426 (p=0.0028) | 0.3426 (p=0.0028) | 0.3568 (p=0.0020) | 0.3426 (p=0.0028) | 0.3426 (p=0.0028) | 0.3426 (p=0.0028) | 0.3426 (p=0.0028) | 0.3426 (p=0.0028) | 0.3426 (p=0.0028) | 0.3426 (p=0.0028) | 0.3426 (p=0.0028) | 0.3426 (p=0.0028) | 0.3426 (p=0.0028) | 0.3426 (p=0.0028) |
| 28 | 0.4722 (p<0.0001) | 0.4478 (p=0.0001) | 0.4722 (p<0.0001) | 0.4707 (p<0.0001) | 0.4460 (p=0.0001) | 0.4722 (p<0.0001) | 0.4707 (p<0.0001) | 0.4307 (p=0.0002) | 0.4707 (p<0.0001) | 0.4707 (p<0.0001) | 0.4722 (p<0.0001) | 0.4707 (p<0.0001) | 0.4707 (p<0.0001) | 0.4707 (p<0.0001) | 0.4707 (p<0.0001) |
| 29 | 0.4474 (p=0.0001) | 0.4477 (p=0.0001) | 0.4488 (p=0.0001) | 0.4474 (p=0.0001) | 0.4477 (p=0.0001) | 0.4488 (p=0.0001) | 0.4474 (p=0.0001) | 0.4491 (p=0.0001) | 0.4477 (p=0.0001) | 0.4488 (p=0.0001) | 0.4491 (p=0.0001) | 0.4477 (p=0.0001) | 0.4488 (p=0.0001) | 0.4491 (p=0.0001) | 0.4491 (p=0.0001) |
| 30 | 0.3417 (p=0.0029) | 0.2840 (p=0.0115) | 0.2683 (p=0.0160) | 0.2607 (p=0.0168) | 0.2864 (p=0.0109) | 0.2787 (p=0.0129) | 0.2689 (p=0.0139) | 0.2157 (p=0.0435) | 0.1771 (p=0.0670) | 0.1726 (p=0.0330) | 0.2298 (p=0.0335) | 0.2056 (p=0.0286) | 0.2391 (p=0.0279) | 0.1570 (p=0.0903) | 0.1635 (p=0.0801) |
| 31 | 0.2826 (p=0.0118) | 0.2834 (p=0.0116) | 0.2826 (p=0.0118) | 0.3103 (p=0.0060) | 0.2834 (p=0.0116) | 0.1943 (p=0.0678) | 0.2493 (p=0.0216) | 0.2939 (p=0.0092) | 0.3157 (p=0.0056) | 0.2969 (p=0.0082) | 0.2283 (p=0.0320) | 0.3088 (p=0.0065) | 0.2914 (p=0.0093) | 0.3077 (p=0.0067) | 0.3026 (p=0.0076) |
| 32 | 0.3644 (p=0.0015) | 0.4917 (p<0.0001) | 0.3957 (p=0.0006) | 0.3957 (p=0.0006) | 0.4490 (p=0.0001) | 0.3809 (p=0.0010) | 0.3874 (p=0.0008) | 0.4985 (p<0.0001) | 0.4905 (p<0.0001) | 0.3957 (p=0.0006) | 0.4521 (p=0.0001) | 0.4445 (p=0.0001) | 0.3874 (p=0.0008) | 0.4957 (p<0.0001) | 0.4496 (p=0.0001) |
| 33 | 0.4745 (p<0.0001) | 0.3866 (p=0.0008) | 0.4745 (p<0.0001) | 0.4777 (p<0.0001) | 0.3603 (p=0.0017) | 0.4745 (p<0.0001) | 0.4777 (p<0.0001) | 0.4298 (p=0.0002) | 0.4440 (p=0.0001) | 0.4777 (p<0.0001) | 0.4216 (p=0.0003) | 0.4389 (p=0.0001) | 0.4777 (p<0.0001) | 0.4562 (p=0.0001) | 0.4792 (p=0.0001) |
| 34 | 0.1528 (p=0.1140) | 0.1528 (p=0.1140) | 0.1528 (p=0.1140) | 0.1528 (p=0.1140) | 0.1528 (p=0.1140) | 0.1528 (p=0.1140) | 0.1528 (p=0.1140) | 0.1528 (p=0.1140) | 0.1528 (p=0.1140) | 0.1528 (p=0.1140) | 0.1528 (p=0.1140) | 0.1528 (p=0.1140) | 0.1528 (p=0.1140) | 0.1528 (p=0.1140) | 0.1528 (p=0.1140) |
| 35 | 0.4976 (p<0.0001) | 0.4995 (p<0.0001) | 0.4972 (p<0.0001) | 0.4976 (p<0.0001) | 0.4995 (p<0.0001) | 0.4972 (p<0.0001) | 0.4976 (p<0.0001) | 0.4974 (p<0.0001) | 0.4995 (p<0.0001) | 0.4972 (p<0.0001) | 0.4974 (p<0.0001) | 0.4995 (p<0.0001) | 0.4972 (p<0.0001) | 0.4974 (p<0.0001) | 0.4974 (p<0.0001) |
| 36 | 0.4864 (p<0.0001) | 0.4847 (p<0.0001) | 0.4856 (p<0.0001) | 0.4856 (p<0.0001) | 0.4856 (p<0.0001) | 0.4864 (p<0.0001) | 0.4864 (p<0.0001) | 0.4847 (p<0.0001) | 0.4847 (p<0.0001) | 0.4856 (p<0.0001) | 0.4856 (p<0.0001) | 0.4856 (p<0.0001) | 0.4864 (p<0.0001) | 0.4847 (p<0.0001) | 0.4856 (p<0.0001) |
| 37 | 0.2843 (p=0.0114) | 0.4269 (p=0.0002) | 0.2847 (p=0.0113) | 0.2843 (p=0.0114) | 0.3983 (p=0.0006) | 0.2847 (p=0.0113) | 0.2843 (p=0.0114) | 0.4093 (p=0.0004) | 0.4283 (p=0.0002) | 0.2847 (p=0.0113) | 0.3995 (p=0.0005) | 0.3963 (p=0.0006) | 0.2847 (p=0.0113) | 0.4120 (p=0.0004) | 0.3891 (p=0.0007) |
| 38 | 0.3251 (p=0.0044) | 0.3804 (p=0.0010) | 0.3804 (p=0.0010) | 0.3804 (p=0.0010) | 0.3804 (p=0.0010) | 0.3804 (p=0.0010) | 0.3804 (p=0.0010) | 0.3804 (p=0.0010) | 0.3804 (p=0.0010) | 0.3804 (p=0.0010) | 0.3804 (p=0.0010) | 0.3804 (p=0.0010) | 0.3804 (p=0.0010) | 0.3804 (p=0.0010) | 0.3804 (p=0.0010) |
| 39 | 0.3441 (p=0.0027) | 0.3441 (p=0.0027) | 0.3441 (p=0.0027) | 0.3441 (p=0.0027) | 0.3441 (p=0.0027) | 0.3441 (p=0.0027) | 0.3441 (p=0.0027) | 0.3441 (p=0.0027) | 0.3441 (p=0.0027) | 0.3441 (p=0.0027) | 0.3441 (p=0.0027) | 0.3441 (p=0.0027) | 0.3441 (p=0.0027) | 0.3441 (p=0.0027) | 0.3441 (p=0.0027) |
| 40 | 0.2990 (p=0.0082) | 0.4175 (p=0.0003) | 0.2804 (p=0.0124) | 0.2990 (p=0.0082) | 0.4141 (p=0.0003) | 0.2990 (p=0.0082) | 0.2990 (p=0.0082) | 0.3814 (p=0.0009) | 0.3958 (p=0.0007) | 0.2990 (p=0.0082) | 0.3990 (p=0.0005) | 0.3923 (p=0.0002) | 0.2990 (p=0.0082) | 0.3825 (p=0.0009) | 0.4087 (p=0.0004) |
| 41 | 0.3671 (p=0.0014) | 0.4163 (p=0.0003) | 0.4479 (p=0.0001) | 0.4409 (p=0.0001) | 0.4124 (p=0.0004) | 0.4392 (p=0.0001) | 0.3705 (p=0.0015) | 0.4550 (p=0.0001) | 0.4409 (p=0.0001) | 0.4510 (p=0.0001) | 0.3866 (p=0.0008) | 0.4025 (p=0.0006) | 0.4357 (p=0.0003) | 0.4399 (p=0.0001) | 0.4239 (p=0.0009) |
| 42 | 0.3450 (p=0.0026) | 0.3450 (p=0.0026) | 0.3450 (p=0.0026) | 0.3450 (p=0.0026) | 0.3363 (p=0.0033) | 0.3450 (p=0.0026) | 0.3450 (p=0.0026) | 0.3450 (p=0.0026) | 0.3450 (p=0.0026) | 0.3450 (p=0.0026) | 0.3450 (p=0.0026) | 0.3450 (p=0.0026) | 0.3450 (p=0.0026) | 0.3450 (p=0.0026) | 0.3450 (p=0.0026) |
| 43 | 0.3901 (p=0.0007) | 0.3917 (p=0.0007) | 0.3913 (p=0.0007) | 0.3901 (p=0.0007) | 0.3917 (p=0.0007) | 0.3913 (p=0.0007) | 0.3901 (p=0.0007) | 0.3929 (p=0.0007) | 0.3917 (p=0.0007) | 0.3913 (p=0.0007) | 0.3929 (p=0.0007) | 0.3917 (p=0.0007) | 0.3913 (p=0.0007) | 0.3929 (p=0.0007) | 0.3929 (p=0.0007) |
| 44 | 0.2587 (p=0.0195) | 0.2587 (p=0.0195) | 0.2579 (p=0.0198) | 0.2587 (p=0.0195) | 0.2587 (p=0.0195) | 0.2579 (p=0.0198) | 0.2587 (p=0.0195) | 0.2579 (p=0.0198) | 0.2587 (p=0.0195) | 0.2579 (p=0.0198) | 0.2579 (p=0.0198) | 0.2587 (p=0.0195) | 0.2579 (p=0.0198) | 0.2579 (p=0.0198) | 0.2579 (p=0.0198) |
| 45 | 0.4396 (p=0.0001) | 0.4094 (p=0.0004) | 0.4593 (p=0.0001) | 0.4570 (p=0.0001) | 0.4061 (p=0.0004) | 0.4599 (p=0.0001) | 0.4186 (p=0.0002) | 0.4484 (p=0.0001) | 0.4267 (p=0.0002) | 0.4573 (p=0.0001) | 0.3746 (p=0.0011) | 0.3637 (p=0.0014) | 0.4290 (p=0.0001) | 0.4367 (p=0.0001) | 0.4410 (p=0.0001) |
| 46 | 0.4174 (p=0.0003) | 0.4207 (p=0.0003) | 0.4037 (p=0.0005) | 0.3726 (p=0.0013) | 0.4521 (p=0.0001) | 0.4344 (p=0.0002) | 0.4162 (p=0.0003) | 0.4600 (p=0.0001) | 0.3888 (p=0.0003) | 0.3997 (p=0.0005) | 0.4777 (p<0.0001) | 0.4504 (p=0.0001) | 0.4347 (p=0.0002) | 0.4509 (p=0.0001) | 0.4674 (p<0.0001) |
| 47 | 0.3534 (p=0.0021) | 0.3534 (p=0.0021) | 0.3534 (p=0.0021) | 0.3534 (p=0.0021) | 0.3534 (p=0.0021) | 0.3534 (p=0.0021) | 0.3534 (p=0.0021) | 0.3534 (p=0.0021) | 0.3534 (p=0.0021) | 0.3534 (p=0.0021) | 0.3534 (p=0.0021) | 0.3534 (p=0.0021) | 0.3534 (p=0.0021) | 0.3534 (p=0.0021) | 0.3534 (p=0.0021) |
| 48 | 0.4168 (p=0.0003) | 0.4411 (p=0.0001) | 0.3929 (p=0.0007) | 0.4354 (p=0.0002) | 0.4410 (p=0.0001) | 0.3848 (p=0.0008) | 0.4281 (p=0.0002) | 0.4232 (p=0.0002) | 0.4392 (p=0.0001) | 0.3928 (p=0.0007) | 0.4245 (p=0.0002) | 0.4385 (p=0.0002) | 0.3839 (p=0.0009) | 0.4184 (p=0.0003) | 0.4192 (p=0.0003) |
| 49 | 0.2788 (p=0.0129) | 0.3734 (p=0.0012) | 0.3490 (p=0.0024) | 0.3570 (p=0.0019) | 0.3513 (p=0.0022) | 0.2407 (p=0.0277) | 0.2824 (p=0.0094) | 0.3761 (p=0.0011) | 0.3876 (p=0.0008) | 0.2623 (p=0.0239) | 0.3155 (p=0.0056) | 0.3296 (p=0.0030) | 0.2433 (p=0.0249) | 0.3783 (p=0.0009) | 0.2275 (p=0.0291) |
| 50 | 0.3664 (p=0.0015) | 0.3871 (p=0.0008) | 0.3771 (p=0.0011) | 0.3304 (p=0.0024) | 0.3914 (p=0.0007) | 0.3828 (p=0.0009) | 0.3563 (p=0.0019) | 0.4022 (p=0.0005) | 0.3324 (p=0.0036) | 0.3304 (p=0.0038) | 0.4011 (p=0.0005) | 0.3756 (p=0.0011) | 0.3706 (p=0.0012) | 0.3818 (p=0.0036) | 0.3872 (p=0.0008) |
| 51 | 0.4375 (p=0.0002) | 0.3113 (p=0.0061) | 0.3611 (p=0.0017) | 0.4397 (p=0.0001) | 0.3524 (p=0.0021) | 0.3881 (p=0.0008) | 0.4376 (p=0.0001) | 0.2923 (p=0.0096) | 0.3502 (p=0.0025) | 0.4390 (p=0.0001) | 0.3513 (p=0.0022) | 0.3909 (p=0.0007) | 0.4369 (p=0.0002) | 0.2934 (p=0.0087) | 0.3402 (p=0.0028) |
| 52 | 0.2241 (p=0.0375) | 0.2241 (p=0.0375) | 0.2615 (p=0.0185) | 0.2241 (p=0.0375) | 0.2241 (p=0.0375) | 0.2241 (p=0.0375) | 0.2241 (p=0.0375) | 0.2241 (p=0.0375) | 0.2241 (p=0.0375) | 0.2241 (p=0.0375) | 0.2241 (p=0.0375) | 0.2241 (p=0.0375) | 0.2241 (p=0.0375) | 0.2241 (p=0.0375) | 0.2241 (p=0.0375) |
| 53 | 0.2288 (p=0.0345) | 0.2288 (p=0.0345) | 0.2536 (p=0.0216) | 0.2273 (p=0.0232) | 0.2288 (p=0.0345) | 0.2288 (p=0.0345) | 0.2273 (p=0.0352) | 0.2288 (p=0.0345) | 0.2273 (p=0.0352) | 0.2273 (p=0.0214) | 0.2288 (p=0.0345) | 0.2273 (p=0.0352) | 0.2273 (p=0.0352) | 0.2273 (p=0.0352) | 0.2273 (p=0.0352) |
| 54 | 0.3691 (p=0.0013) | 0.3691 (p=0.0013) | 0.3677 (p=0.0014) | 0.3702 (p=0.0013) | 0.3691 (p=0.0013) | 0.3677 (p=0.0014) | 0.3702 (p=0.0013) | 0.3677 (p=0.0014) | 0.3702 (p=0.0013) | 0.3689 (p=0.0014) | 0.3677 (p=0.0014) | 0.3702 (p=0.0013) | 0.3689 (p=0.0014) | 0.3689 (p=0.0014) | 0.3689 (p=0.0014) |
| 55 | 0.3672 (p=0.0014) | 0.3179 (p=0.0052) | 0.3848 (p=0.0008) | 0.3672 (p=0.0014) | 0.3282 (p=0.0041) | 0.3982 (p=0.0006) | 0.3672 (p=0.0014) | 0.3343 (p=0.0035) | 0.3174 (p=0.0054) | 0.3814 (p=0.0009) | 0.3428 (p=0.0028) | 0.3288 (p=0.0041) | 0.3970 (p=0.0006) | 0.3344 (p=0.0035) | 0.3433 (p=0.0028) |
| 56 | 0.3636 (p=0.0016) | 0.2980 (p=0.0084) | 0.3523 (p=0.0022) | 0.3523 (p=0.0022) | 0.3028 (p=0.0075) | 0.3523 (p=0.0022) | 0.3523 (p=0.0022) | 0.2923 (p=0.0095) | 0.3108 (p=0.0060) | 0.3523 (p=0.0022) | 0.2956 (p=0.0089) | 0.3143 (p=0.0055) | 0.3523 (p=0.0022) | 0.3029 (p=0.0072) | 0.3050 (p=0.0069) |
| 57 | 0.2869 (p=0.0108) | 0.3021 (p=0.0076) | 0.2842 (p=0.0114) | 0.2869 (p=0.0108) | 0.2921 (p=0.0096) | 0.2772 (p=0.0133) | 0.2869 (p=0.0108) | 0.3060 (p=0.0070) | 0.3154 (p=0.0048) | 0.3031 (p=0.0061) | 0.2979 (p=0.0084) | 0.3063 (p=0.0058) | 0.2959 (p=0.0068) | 0.3143 (p=0.0051) | 0.3088 (p=0.0056) |
| 58 | 0.1429 (p=0.1301) | 0.5113 (p<0.0001) | 0.4454 (p=0.0001) | 0.4119 (p=0.0003) | 0.3355 (p=0.0034) | 0.2189 (p=0.0409) | 0.3600 (p=0.0017) | 0.4861 (p<0.0001) | 0.4504 (p=0.0001) | 0.3455 (p=0.0053) | 0.3073 (p=0.0067) | 0.3930 (p=0.0005) | 0.2016 (p=0.0849) | 0.3408 (p=0.0075) | 0.2961 (p=0.0162) |
| 59 | 0.3029 (p=0.0075) | 0.3779 (p=0.0010) | 0.3420 (p=0.0028) | 0.3404 (p=0.0129) | 0.3796 (p=0.0010) | 0.3809 (p=0.0010) | 0.2997 (p=0.0078) | 0.3993 (p=0.0005) | 0.3856 (p=0.0054) | 0.3385 (p=0.0027) | 0.3872 (p=0.0008) | 0.3697 (p=0.0013) | 0.3809 (p=0.0010) | 0.3871 (p=0.0019) | 0.3759 (p=0.0011) |
| 60 | 0.3432 (p=0.0028) | 0.3586 (p=0.0018) | 0.3915 (p=0.0007) | 0.3432 (p=0.0028) | 0.3515 (p=0.0022) | 0.3432 (p=0.0028) | 0.3432 (p=0.0028) | 0.3623 (p=0.0016) | 0.3840 (p=0.0009) | 0.3432 (p=0.0028) | 0.3576 (p=0.0019) | 0.3669 (p=0.0014) | 0.3432 (p=0.0028) | 0.3884 (p=0.0008) | 0.3423 (p=0.0028) |
| 61 | 0.3860 (p=0.0008) | 0.3987 (p=0.0006) | 0.3890 (p=0.0007) | 0.4278 (p=0.0009) | 0.3873 (p=0.0008) | 0.3771 (p=0.0011) | 0.3416 (p=0.0006) | 0.3789 (p=0.0010) | 0.4175 (p=0.0003) | 0.3958 (p=0.0006) | 0.2985 (p=0.0083) | 0.3005 (p=0.0003) | 0.3958 (p=0.0006) | 0.4004 (p=0.0005) | 0.2924 (p=0.0005) |
| 62 | 0.2390 (p=0.0286) | 0.1877 (p=0.0687) | 0.2188 (p=0.0412) | 0.2325 (p=0.0301) | 0.2022 (p=0.0546) | 0.2523 (p=0.0222) | 0.2587 (p=0.0183) | 0.1939 (p=0.0624) | 0.2209 (p=0.0409) | 0.2379 (p=0.0282) | 0.2612 (p=0.0185) | 0.2315 (p=0.0338) | 0.2655 (p=0.0164) | 0.2259 (p=0.0360) | 0.2674 (p=0.0163) |
| 63 | 0.3344 (p=0.0035) | 0.3816 (p=0.0009) | 0.3693 (p=0.0013) | 0.3717 (p=0.0012) | 0.3734 (p=0.0012) | 0.3732 (p=0.0012) | 0.3717 (p=0.0012) | 0.3861 (p=0.0008) | 0.3717 (p=0.0012) | 0.3732 (p=0.0012) | 0.3732 (p=0.0012) | 0.3717 (p=0.0012) | 0.3732 (p=0.0012) | 0.3732 (p=0.0012) | 0.3732 (p=0.0012) |
| 64 | 0.2180 (p=0.0418) | 0.2111 (p=0.0470) | 0.2180 (p=0.0418) | 0.2734 (p=0.0151) | 0.2138 (p=0.0449) | 0.2180 (p=0.0418) | 0.2180 (p=0.0418) | 0.2112 (p=0.0470) | 0.2350 (p=0.0309) | 0.2636 (p=0.0208) | 0.2146 (p=0.0443) | 0.2083 (p=0.0508) | 0.2180 (p=0.0418) | 0.2071 (p=0.0542) | 0.2105 (p=0.0512) |
| 65 | 0.5025 (p<0.0001) | 0.4448 (p=0.0001) | 0.4268 (p=0.0002) | 0.5028 (p<0.0001) | 0.4133 (p=0.0003) | 0.5025 (p<0.0001) | 0.5028 (p<0.0001) | 0.4459 (p=0.0001) | 0.4602 (p=0.0001) | 0.4430 (p=0.0001) | 0.3940 (p=0.0006) | 0.5007 (p<0.0001) | 0.5028 (p<0.0001) | 0.4574 (p=0.0001) | 0.5007 (p<0.0001) |
| 66 | 0.3502 (p=0.0023) | 0.4087 (p=0.0004) | 0.4462 (p=0.0001) | 0.4047 (p=0.0004) | 0.3884 (p=0.0008) | 0.3891 (p=0.0007) | 0.3578 (p=0.0018) | 0.4201 (p=0.0003) | 0.3680 (p=0.0011) | 0.4163 (p=0.0003) | 0.3951 (p=0.0006) | 0.3624 (p=0.0014) | 0.3742 (p=0.0012) | 0.3912 (p=0.0006) | 0.3696 (p=0.0012) |
| 67 | 0.3941 (p=0.0006) | 0.3952 (p=0.0006) | 0.4397 (p=0.0001) | 0.4703 (p<0.0001) | 0.3684 (p=0.0014) | 0.3892 (p=0.0007) | 0.3964 (p<0.0001) | 0.3983 (p=0.0006) | 0.4093 (p=0.0004) | 0.4686 (p<0.0001) | 0.3718 (p=0.0012) | 0.3824 (p=0.0009) | 0.4686 (p<0.0001) | 0.4090 (p=0.0004) | 0.3809 (p=0.0010) |
| 68 | 0.2262 (p=0.0362) | 0.2857 (p=0.0111) | 0.2737 (p=0.0143) | 0.2420 (p=0.0346) | 0.2262 (p=0.0362) | 0.2262 (p=0.0362) | 0.2262 (p=0.0362) | 0.2286 (p=0.0346) | 0.2286 (p=0.0346) | 0.2286 (p=0.0346) | 0.2262 (p=0.0362) | 0.2262 (p=0.0362) | 0.2262 (p=0.0362) | 0.2286 (p=0.0346) | 0.2262 (p=0.0362) |
| 69 | 0.2135 (p=0.0451) | 0.2888 (p=0.0103) | 0.2535 (p=0.0216) | 0.1565 (p=0.1043) | 0.2135 (p=0.0451) | 0.2135 (p=0.0451) | 0.1449 (p=0.1253) | 0.3040 (p=0.0073) | 0.2165 (p=0.0403) | 0.1716 (p=0.0820) | 0.2596 (p=0.0192) | 0.2037 (p=0.0499) | 0.1620 (p=0.0975) | 0.2276 (p=0.0328) | 0.2145 (p=0.0412) |
| 70 | 0.6206 (p<0.0001) | 0.6205 (p<0.0001) | 0.6209 (p<0.0001) | 0.6224 (p<0.0001) | 0.6206 (p<0.0001) | 0.6210 (p<0.0001) | 0.6227 (p<0.0001) | 0.6209 (p<0.0001) | 0.6224 (p<0.0001) | 0.6229 (p<0.0001) | 0.6210 (p<0.0001) | 0.6227 (p<0.0001) | 0.6231 (p<0.0001) | 0.6229 (p<0.0001) | 0.6231 (p<0.0001) |
| 71 | 0.3140 (p=0.0058) | 0.3140 (p=0.0058) | 0.3140 (p=0.0058) | 0.3140 (p=0.0058) | 0.3140 (p=0.0058) | 0.3140 (p=0.0058) | 0.3140 (p=0.0058) | 0.3140 (p=0.0058) | 0.3140 (p=0.0058) | 0.3140 (p=0.0058) | 0.3140 (p=0.0058) | 0.3140 (p=0.0058) | 0.3140 (p=0.0058) | 0.3140 (p=0.0058) | 0.3140 (p=0.0058) |
| 72 | 0.4904 (p<0.0001) | 0.4869 (p<0.0001) | 0.5244 (p<0.0001) | 0.4916 (p<0.0001) | 0.4869 (p<0.0001) | 0.4718 (p<0.0001) | 0.4916 (p<0.0001) | 0.4881 (p<0.0001) | 0.4882 (p<0.0001) | 0.5404 (p<0.0001) | 0.4881 (p<0.0001) | 0.4882 (p<0.0001) | 0.4927 (p<0.0001) | 0.4893 (p<0.0001) | 0.4893 (p<0.0001) |
| 73 | 0.2377 (p=0.0293) | 0.2377 (p=0.0293) | 0.2377 (p=0.0293) | 0.2377 (p=0.0293) | 0.2377 (p=0.0293) | 0.2377 (p=0.0293) | 0.2377 (p=0.0293) | 0.3085 (p=0.0066) | 0.2377 (p=0.0293) | 0.2377 (p=0.0293) | 0.2377 (p=0.0293) | 0.2377 (p=0.0293) | 0.2377 (p=0.0293) | 0.2377 (p=0.0293) | 0.2377 (p=0.0293) |
| 74 | 0.4427 (p=0.0001) | 0.3863 (p=0.0008) | 0.4414 (p=0.0001) | 0.4414 (p=0.0001) | 0.4149 (p=0.0003) | 0.4427 (p=0.0001) | 0.4427 (p=0.0001) | 0.3863 (p=0.0008) | 0.3901 (p=0.0007) | 0.4414 (p=0.0001) | 0.4143 (p=0.0003) | 0.4150 (p=0.0003) | 0.4427 (p=0.0001) | 0.3925 (p=0.0007) | 0.4148 (p=0.0003) |
| 75 | 0.0717 (p=0.2866) | 0.1303 (p=0.1524) | 0.1305 (p=0.1520) | 0.1031 (p=0.2089) | 0.0998 (p=0.2171) | 0.1031 (p=0.2089) | 0.1031 (p=0.2089) | 0.1460 (p=0.1249) | 0.1504 (p=0.1159) | 0.1031 (p=0.2089) | 0.1244 (p=0.1636) | 0.1258 (p=0.1564) | 0.1031 (p=0.2089) | 0.1455 (p=0.1261) | 0.1311 (p=0.1397) |
| 76 | 0.3502 (p=0.0023) | 0.3619 (p=0.0016) | 0.3050 (p=0.0071) | 0.3502 (p=0.0023) | 0.3613 (p=0.0017) | 0.3046 (p=0.0072) | 0.3312 (p=0.0068) | 0.3381 (p=0.0031) | 0.3651 (p=0.0014) | 0.3150 (p=0.0056) | 0.3360 (p=0.0033) | 0.3632 (p=0.0015) | 0.3128 (p=0.0058) | 0.3413 (p=0.0030) | 0.3415 (p=0.0028) |
| 77 | 0.4507 (p=0.0001) | 0.3721 (p=0.0012) | 0.4507 (p=0.0001) | 0.4506 (p=0.0001) | 0.3730 (p=0.0012) | 0.4507 (p=0.0001) | 0.4506 (p=0.0001) | 0.3870 (p=0.0008) | 0.3850 (p=0.0007) | 0.4506 (p=0.0001) | 0.3812 (p=0.0009) | 0.3833 (p=0.0007) | 0.4506 (p=0.0001) | 0.3955 (p=0.0005) | 0.3891 (p=0.0006) |
| 78 | 0.1987 (p=0.0578) | 0.1987 (p=0.0578) | 0.1927 (p=0.0635) | 0.2271 (p=0.0336) | 0.1987 (p=0.0578) | 0.1927 (p=0.0635) | 0.1987 (p=0.0578) | 0.1927 (p=0.0635) | 0.2412 (p=0.0280) | 0.1927 (p=0.0635) | 0.1927 (p=0.0635) | 0.1987 (p=0.0578) | 0.1927 (p=0.0635) | 0.2398 (p=0.0285) | 0.1927 (p=0.0635) |
| 79 | 0.3580 (p=0.0018) | 0.3568 (p=0.0019) | 0.3580 (p=0.0018) | 0.3401 (p=0.0029) | 0.3580 (p=0.0018) | 0.3580 (p=0.0018) | 0.3296 (p=0.0038) | 0.3580 (p=0.0018) | 0.3737 (p=0.0012) | 0.3498 (p=0.0022) | 0.3580 (p=0.0018) | 0.3589 (p=0.0017) | 0.3365 (p=0.0032) | 0.3775 (p=0.0011) | 0.3622 (p=0.0016) |
| 80 | 0.2887 (p=0.0103) | 0.4223 (p=0.0003) | 0.3479 (p=0.0024) | 0.3504 (p=0.0023) | 0.4141 (p=0.0003) | 0.3479 (p=0.0024) | 0.3504 (p=0.0023) | 0.4181 (p=0.0003) | 0.4118 (p=0.0004) | 0.3504 (p=0.0023) | 0.4101 (p=0.0004) | 0.4070 (p=0.0004) | 0.3504 (p=0.0023) | 0.4107 (p=0.0004) | 0.4065 (p=0.0004) |
| 81 | 0.4430 (p=0.0001) | 0.4890 (p<0.0001) | 0.4413 (p=0.0001) | 0.4181 (p=0.0003) | 0.4886 (p<0.0001) | 0.4413 (p=0.0001) | 0.4296 (p=0.0002) | 0.4501 (p=0.0001) | 0.4426 (p=0.0001) | 0.4300 (p=0.0002) | 0.4515 (p=0.0001) | 0.4454 (p=0.0001) | 0.4300 (p=0.0002) | 0.4204 (p=0.0002) | 0.4238 (p=0.0002) |
| 82 | 0.3426 (p=0.0028) | 0.3444 (p=0.0027) | 0.3431 (p=0.0028) | 0.3332 (p=0.0024) | 0.3439 (p=0.0027) | 0.3426 (p=0.0028) | 0.3229 (p=0.0036) | 0.3444 (p=0.0027) | 0.3441 (p=0.0026) | 0.3428 (p=0.0027) | 0.3439 (p=0.0027) | 0.3435 (p=0.0026) | 0.3422 (p=0.0027) | 0.3441 (p=0.0026) | 0.3435 (p=0.0026) |
| 83 | 0.2058 (p=0.0514) | 0.2419 (p=0.0270) | 0.2438 (p=0.0261) | 0.2419 (p=0.0270) | 0.2419 (p=0.0270) | 0.2244 (p=0.0373) | 0.2085 (p=0.0486) | 0.2438 (p=0.0261) | 0.2419 (p=0.0270) | 0.2438 (p=0.0261) | 0.2438 (p=0.0261) | 0.2419 (p=0.0270) | 0.2438 (p=0.0371) | 0.2438 (p=0.0261) | 0.2438 (p=0.0261) |
| 84 | 0.1893 (p=0.0670) | 0.3693 (p=0.0013) | 0.3683 (p=0.0014) | 0.3682 (p=0.0014) | 0.2463 (p=0.0249) | 0.1940 (p=0.0622) | 0.1907 (p=0.0652) | 0.3683 (p=0.0014) | 0.3682 (p=0.0014) | 0.3683 (p=0.0014) | 0.2325 (p=0.0322) | 0.2289 (p=0.0334) | 0.1938 (p=0.0625) | 0.3683 (p=0.0014) | 0.2111 (p=0.0456) |
| 85 | 0.5717 (p<0.0001) | 0.5570 (p<0.0001) | 0.4801 (p<0.0001) | 0.5712 (p<0.0001) | 0.5721 (p<0.0001) | 0.4856 (p<0.0001) | 0.5716 (p<0.0001) | 0.4828 (p<0.0001) | 0.5587 (p<0.0001) | 0.4822 (p<0.0001) | 0.4856 (p<0.0001) | 0.5654 (p<0.0001) | 0.4887 (p<0.0001) | 0.4873 (p<0.0001) | 0.4941 (p<0.0001) |
| 86 | 0.3215 (p=0.0048) | 0.3803 (p=0.0010) | 0.3214 (p=0.0048) | 0.3214 (p=0.0048) | 0.3215 (p=0.0048) | 0.3215 (p=0.0048) | 0.3215 (p=0.0048) | 0.3214 (p=0.0048) | 0.3214 (p=0.0048) | 0.3214 (p=0.0048) | 0.3215 (p=0.0048) | 0.3215 (p=0.0048) | 0.3215 (p=0.0048) | 0.3214 (p=0.0048) | 0.3215 (p=0.0048) |
| 87 | 0.4693 (p<0.0001) | 0.4430 (p=0.0001) | 0.4278 (p=0.0002) | 0.4693 (p<0.0001) | 0.4709 (p<0.0001) | 0.4337 (p=0.0002) | 0.4693 (p<0.0001) | 0.4387 (p=0.0001) | 0.4709 (p<0.0001) | 0.4693 (p=0.0007) | 0.4468 (p=0.0001) | 0.4709 (p<0.0001) | 0.4693 (p<0.0001) | 0.4251 (p=0.0006) | 0.4695 (p<0.0001) |
| 88 | 0.2585 (p=0.0196) | 0.2589 (p=0.0194) | 0.2589 (p=0.0194) | 0.2323 (p=0.0302) | 0.2585 (p=0.0196) | 0.2585 (p=0.0196) | 0.2356 (p=0.0285) | 0.2589 (p=0.0194) | 0.2238 (p=0.0554) | 0.2292 (p=0.0320) | 0.2585 (p=0.0196) | 0.2262 (p=0.0452) | 0.2319 (p=0.0305) | 0.2315 (p=0.0318) | 0.2339 (p=0.0302) |
| 89 | 0.2870 (p=0.0108) | 0.2879 (p=0.0105) | 0.2870 (p=0.0108) | 0.2870 (p=0.0108) | 0.2879 (p=0.0105) | 0.2870 (p=0.0108) | 0.2870 (p=0.0108) | 0.2879 (p=0.0105) | 0.2879 (p=0.0105) | 0.2870 (p=0.0108) | 0.2879 (p=0.0105) | 0.2879 (p=0.0105) | 0.2870 (p=0.0108) | 0.2879 (p=0.0105) | 0.2879 (p=0.0105) |
| 90 | 0.2681 (p=0.0161) | 0.2681 (p=0.0161) | 0.2681 (p=0.0161) | 0.2575 (p=0.0335) | 0.2681 (p=0.0161) | 0.2681 (p=0.0161) | 0.2646 (p=0.0228) | 0.2681 (p=0.0161) | 0.2687 (p=0.0207) | 0.2607 (p=0.0247) | 0.2681 (p=0.0161) | 0.2733 (p=0.0189) | 0.2676 (p=0.0216) | 0.2701 (p=0.0201) | 0.2762 (p=0.0179) |
| 91 | 0.2730 (p=0.0145) | 0.2730 (p=0.0145) | 0.3363 (p=0.0033) | 0.3078 (p=0.0061) | 0.2730 (p=0.0145) | 0.2730 (p=0.0145) | 0.2730 (p=0.0145) | 0.2730 (p=0.0145) | 0.2730 (p=0.0145) | 0.3325 (p=0.0037) | 0.2730 (p=0.0145) | 0.2730 (p=0.0145) | 0.2730 (p=0.0145) | 0.2730 (p=0.0145) | 0.2730 (p=0.0145) |
| 92 | 0.3567 (p=0.0019) | 0.3579 (p=0.0018) | 0.3556 (p=0.0020) | 0.3569 (p=0.0020) | 0.3579 (p=0.0018) | 0.3556 (p=0.0020) | 0.3569 (p=0.0020) | 0.3556 (p=0.0020) | 0.3581 (p=0.0019) | 0.3558 (p=0.0020) | 0.3556 (p=0.0020) | 0.3581 (p=0.0019) | 0.3378 (p=0.0035) | 0.3558 (p=0.0020) | 0.3558 (p=0.0020) |
| 93 | 0.3306 (p=0.0038) | 0.3918 (p=0.0007) | 0.3306 (p=0.0038) | 0.3306 (p=0.0038) | 0.3690 (p=0.0013) | 0.3306 (p=0.0038) | 0.3306 (p=0.0038) | 0.3306 (p=0.0038) | 0.3863 (p=0.0007) | 0.3306 (p=0.0038) | 0.3306 (p=0.0038) | 0.3734 (p=0.0012) | 0.3306 (p=0.0038) | 0.3940 (p=0.0038) | 0.3306 (p=0.0038) |
| 94 | 0.3354 (p=0.0034) | 0.3326 (p=0.0036) | 0.3354 (p=0.0034) | 0.3081 (p=0.0048) | 0.2975 (p=0.0085) | 0.3354 (p=0.0034) | 0.3359 (p=0.0034) | 0.3209 (p=0.0049) | 0.2995 (p=0.0075) | 0.3359 (p=0.0034) | 0.2982 (p=0.0084) | 0.2916 (p=0.0093) | 0.3359 (p=0.0034) | 0.2942 (p=0.0085) | 0.2879 (p=0.0102) |
| 95 | 0.3935 (p=0.0006) | 0.3935 (p=0.0006) | 0.3935 (p=0.0006) | 0.3216 (p=0.0059) | 0.3935 (p=0.0006) | 0.3935 (p=0.0006) | 0.3197 (p=0.0054) | 0.3935 (p=0.0006) | 0.3924 (p=0.0007) | 0.3200 (p=0.0109) | 0.3935 (p=0.0006) | 0.3924 (p=0.0007) | 0.3924 (p=0.0007) | 0.3924 (p=0.0007) | 0.3924 (p=0.0007) |
| 96 | 0.4237 (p=0.0002) | 0.4868 (p<0.0001) | 0.4237 (p=0.0002) | 0.4237 (p=0.0002) | 0.4237 (p=0.0002) | 0.4237 (p=0.0002) | 0.4237 (p=0.0002) | 0.4237 (p=0.0002) | 0.4237 (p=0.0002) | 0.4237 (p=0.0002) | 0.4237 (p=0.0002) | 0.4237 (p=0.0002) | 0.4237 (p=0.0002) | 0.4237 (p=0.0002) | 0.4237 (p=0.0002) |
| 97 | 0.2839 (p=0.0115) | 0.3455 (p=0.0026) | 0.3094 (p=0.0064) | 0.3149 (p=0.0056) | 0.3063 (p=0.0069) | 0.3094 (p=0.0064) | 0.3149 (p=0.0056) | 0.3130 (p=0.0059) | 0.3199 (p=0.0050) | 0.3154 (p=0.0056) | 0.3130 (p=0.0059) | 0.3199 (p=0.0050) | 0.3154 (p=0.0056) | 0.3192 (p=0.0051) | 0.3192 (p=0.0051) |
| 98 | 0.4702 (p<0.0001) | 0.4165 (p=0.0003) | 0.4685 (p<0.0001) | 0.4662 (p=0.0001) | 0.4411 (p=0.0001) | 0.4685 (p<0.0001) | 0.4662 (p=0.0001) | 0.4149 (p=0.0003) | 0.4071 (p=0.0005) | 0.4656 (p=0.0001) | 0.4352 (p=0.0002) | 0.4311 (p=0.0002) | 0.4656 (p=0.0001) | 0.4138 (p=0.0003) | 0.4295 (p=0.0002) |
| 99 | 0.3217 (p=0.0048) | 0.4252 (p=0.0002) | 0.3540 (p=0.0021) | 0.3540 (p=0.0021) | 0.4026 (p=0.0005) | 0.3540 (p=0.0021) | 0.3540 (p=0.0021) | 0.4197 (p=0.0003) | 0.3629 (p=0.0016) | 0.3540 (p=0.0021) | 0.3629 (p=0.0016) | 0.3629 (p=0.0016) | 0.3540 (p=0.0021) | 0.3629 (p=0.0016) | 0.3629 (p=0.0016) |
| 100 | 0.1659 (p=0.0950) | 0.1998 (p=0.0567) | 0.1653 (p=0.0959) | 0.1659 (p=0.0950) | 0.1850 (p=0.0717) | 0.1653 (p=0.0959) | 0.1659 (p=0.0950) | 0.1995 (p=0.0570) | 0.2026 (p=0.0538) | 0.1653 (p=0.0959) | 0.1943 (p=0.0620) | 0.1883 (p=0.0676) | 0.1653 (p=0.0959) | 0.2003 (p=0.0559) | 0.1956 (p=0.0603) |
