# Supplementary material for: Integrating multiple brain imaging modalities does not boost prediction of subclinical atherosclerosis in midlife adults
Source: Neuroimage Clin. 2022 Jul 29;35:103134. doi: 10.1016/j.nicl.2022.103134 (PMC9421527; doi:10.1016/j.nicl.2022.103134)
Supplement: Supplementary data 3 [file mmc3.docx]

**Supplementary Table 3:** *95% Confidence interval for Pearson correlation coefficient values.*

For each single channel and channel combination, the 95% confidence interval for the Pearson correlation coefficient values from all Monte Carlo data partitions was calculated using 1000 bootstrapped iterations. Channel combinations are indicated numerically with 1 = resting-state FC, 2 = cortical SA, 3 = cortical thickness, 4 = subcortical volume, 5 = FRS. FC = functional connectivity, SA = surface area, FRS = Framingham Risk Score.

| **Single Channel or Channel Combination** | **95% CI** |
| --- | --- |
| resting-state FC | [-0.0375, 0.0096] |
| cortical SA | [0.0959, 0.1402] |
| cortical thickness | [0.1237, 0.1560] |
| subcortical volume | [0.1245, 0.1646] |
| FRS | [0.3739, 0.4058] |
| (1, 2) | [0.0377, 0.0819] |
| (1, 3) | [0.0698, 0.1084] |
| (1, 4) | [0.0025, 0.0467] |
| (2, 3) | [0.0963, 0.1383] |
| (2, 4) | [0.0555, 0.1001] |
| (3, 4) | [0.0857, 0.1175] |
| (1, 2, 3) | [0.0778, 0.1197] |
| (1, 2, 4) | [0.0376, 0.0797] |
| (1, 3, 4) | [0.0689, 0.1042] |
| (2, 3, 4) | [0.0849, 0.1206] |
| (1, 2, 3, 4) | [0.0671, 0.1041] |
| (1, 5) | [0.3261, 0.3653] |
| (2, 5) | [0.3412, 0.3772] |
| (3, 5) | [0.3357, 0.3719] |
| (4, 5) | [0.3359, 0.3735] |
| (1, 2, 5) | [0.3306, 0.3670] |
| (1, 3, 5) | [0.3246, 0.3646] |
| (1, 4, 5) | [0.3246, 0.3640] |
| (2, 3, 5) | [0.3353, 0.3725] |
| (2, 4, 5) | [0.3351, 0.3722] |
| (3, 4, 5) | [0.3273, 0.3656] |
| (1, 2, 3, 5) | [0.3260, 0.3622] |
| (1, 2, 4, 5) | [0.3287, 0.3651] |
| (1, 3, 4, 5) | [0.3243, 0.3651] |
| (2, 3, 4, 5) | [0.3298, 0.3672] |
| (1, 2, 3, 4, 5) | [0.3236, 0.3617] |
