# Supplementary material for: Integrating multiple brain imaging modalities does not boost prediction of subclinical atherosclerosis in midlife adults
Source: Neuroimage Clin. 2022 Jul 29;35:103134. doi: 10.1016/j.nicl.2022.103134 (PMC9421527; doi:10.1016/j.nicl.2022.103134)
Supplement: Supplementary data 4 [file mmc4.docx]

**Supplementary Table 4:** *95% Confidence interval for coefficient of determination, R-squared values.*

For each single channel and channel combination, the 95% confidence interval for the coefficient of determination, R-squared values from all Monte Carlo data partitions was calculated using 1000 bootstrapped iterations. Channel combinations are indicated numerically with 1 = resting-state FC, 2 = cortical SA, 3 = cortical thickness, 4 = subcortical volume, 5 = FRS. FC = functional connectivity, SA = surface area, FRS = Framingham Risk Score.

| **Single Channel or Channel Combination** | **95% CI** |
| --- | --- |
| resting-state FC | [-0.1343, -0.0937] |
| cortical SA | [-0.1271, -0.0819] |
| cortical thickness | [-0.1060, -0.0674] |
| subcortical volume | [-0.3234, -0.2459] |
| FRS | [0.1139, 0.1421] |
| (1, 2) | [-0.0531, -0.0277] |
| (1, 3) | [-0.0411, -0.0178] |
| (1, 4) | [-0.0507, -0.0300] |
| (2, 3) | [-0.0396, -0.0124] |
| (2, 4) | [-0.0467, -0.0217] |
| (3, 4) | [-0.0407, -0.0172] |
| (1, 2, 3) | [-0.0422, -0.0173] |
| (1, 2, 4) | [-0.0511, -0.0278] |
| (1, 3, 4) | [-0.0447, -0.0214] |
| (2, 3, 4) | [-0.0408, -0.0164] |
| (1, 2, 3, 4) | [-0.0450, -0.0211] |
| (1, 5) | [0.0757, 0.1085] |
| (2, 5) | [0.0855, 0.1186] |
| (3, 5) | [0.0804, 0.1132] |
| (4, 5) | [0.0823, 0.1130] |
| (1, 2, 5) | [0.0778, 0.1104] |
| (1, 3, 5) | [0.0694, 0.1049] |
| (1, 4, 5) | [0.0731, 0.1051] |
| (2, 3, 5) | [0.0817, 0.1148] |
| (2, 4, 5) | [0.0810, 0.1131] |
| (3, 4, 5) | [0.0722, 0.1055] |
| (1, 2, 3, 5) | [0.0728, 0.1059] |
| (1, 2, 4, 5) | [0.0758, 0.1079] |
| (1, 3, 4, 5) | [0.0701, 0.1046] |
| (2, 3, 4, 5) | [0.0755, 0.1092] |
| (1, 2, 3, 4, 5) | [0.0709, 0.1042] |
